# Global dominance of *Haloquadratum walsbyi* by a single genomovar with distinct gene content and viral cohorts from close relatives

**DOI:** 10.1093/ismejo/wraf165

**Published:** 2025-08-04

**Authors:** Esteban Bustos-Caparros, Tomeu Viver, Juan F Gago, Juanita R Avontuur, Souad Amiour, Bonnie K Baxter, María E Llames, Mehmet B Mutlu, Aharon Oren, Ana S Ramírez, Matthew B Stott, Stephanus N Venter, Fernando Santos, Josefa Antón, Luis M Rodriguez-R, Rafael Bosch, Brian P Hedlund, Konstantinos T Konstantinidis, Ramon Rossello-Mora

**Affiliations:** Marine Microbiology Group (MMG), Department of Animal and Microbial Biodiversity, Mediterranean Institute for Advanced Studies (IMEDEA, CSIC-UIB), 07190 Esporles, Illes Balears, Spain; Marine Microbiology Group (MMG), Department of Animal and Microbial Biodiversity, Mediterranean Institute for Advanced Studies (IMEDEA, CSIC-UIB), 07190 Esporles, Illes Balears, Spain; Marine Microbiology Group (MMG), Department of Animal and Microbial Biodiversity, Mediterranean Institute for Advanced Studies (IMEDEA, CSIC-UIB), 07190 Esporles, Illes Balears, Spain; Marine Microbiology Group (MMG), Department of Animal and Microbial Biodiversity, Mediterranean Institute for Advanced Studies (IMEDEA, CSIC-UIB), 07190 Esporles, Illes Balears, Spain; Laboratory of Bioengineering, National Higher School of Biotechnology, Ali Mendjeli University Town, E66 P.O. Box. Constantine 25100, Constantine, Algeria; Great Salt Lake Institute, Westminster University, 1840 S 1300 E, Salt Lake City, UT 84105, United States; Laboratorio de Ecología Microbiana Ambiental, Instituto Tecnológico de Chascomús (CONICET-UNSAM), Escuela de Bio y Nanotecnologías (UNSAM), Cam. Circunvalación Km. 8,2, B7130 Chascomús, Buenos Aires, Argentina; Department of Biology, Eskisehir Technical University, 26470 Eskisehir, Turkey; Department of Plant and Environmental Sciences, The Institute of Life Sciences, The Hebrew University of Jerusalem, Edmond J. Safra Campus, Givat Ram, Jerusalem 9190401, Israel; Unidad de Epidemiología y Medicina Preventiva, IUSA, Facultad de Veterinaria, Universidad de Las Palmas de Gran Canaria, C/ Trasmontaña s/n, Arucas, 35413 Canary Islands, Spain; School of Biological Sciences, University of Canterbury, Christchurch, 20 Kirkwood Avenue, Upper Riccarton, 8041 Christchurch, New Zealand; Department of Biochemistry, Genetics and Microbiology, and Forestry and Agricultural Biotechnology Institute (FABI), University of Pretoria, Hatfield, 0028, Pretoria, South Africa; Department of Physiology, Genetics and Microbiology, University of Alicante, 03690 San Vicent del Raspeig, Alicante, Spain; Department of Physiology, Genetics and Microbiology, University of Alicante, 03690 San Vicent del Raspeig, Alicante, Spain; Department of Microbiology and Digital Science Center (DiSC), University of Innsbruck, Innrain 15, 6020 Innsbruck, Austria; Microbiologia, Departament de Biologia, Edifici Guillem Colom, Universitat de les Illes Balears, Campus UIB, 07122 Palma de Mallorca, Illes Balears, Spain; School of Life Sciences, University of Nevada, 4505 S. Maryland Pkwy, Las Vegas, NV 89154-4004, United States; School of Civil and Environmental Engineering, Georgia Institute of Technology, Atlanta, GA 30332, United States; Marine Microbiology Group (MMG), Department of Animal and Microbial Biodiversity, Mediterranean Institute for Advanced Studies (IMEDEA, CSIC-UIB), 07190 Esporles, Illes Balears, Spain

**Keywords:** Haloquadratum walsbyi, osmotic disturbances, metagenomics, time-series, intraspecies diversity, genomovar, halovirus

## Abstract

*Haloquadratum walsbyi* is generally the dominant species in hypersaline ecosystems at salt saturation conditions. Here, we followed the dynamics of its genomovars and associated viruses during recurrent evaporation-dilution disturbances of varying intensities at the mesocosm scale over 813 days. The diversity observed within a single mesocosm was also compared with that in a global-scale inventory of hypersaline environments of thalassohaline origin. The 140 binned metagenome assembled genomes (MAGs) together with the genomes of the (only) two available of *H. walsbyi* isolates grouped into four highly related (98.25% > Average Nucleotide Identity [ANI] > 99.5%) dominant genomovars (intra-genomovar ANI > 99.5%). In mesocosm experiments, moderate disturbances (i.e. recurrent dilution from saturation to 20% salts) enhanced the abundance of the already-dominant genomovar Hqrw1, resulting in reduced intraspecific diversity. This genomovar also dominated in almost all sites sampled around the globe. In contrast, more intense disturbance (i.e. recurrent dilution from saturation to 13% salts) decreased the abundance of Hqrw1 to lower levels than genomovar Hqrw2 by the end of the incubation, which seems to resist better osmotic changes. Further, our results showed that genomovars were followed by their viral cohorts, who play a significant role in the global dominance of the four *H. walsbyi* genomovars and their replacement under unfavorable conditions. We propose that the global dominance of *H. walsbyi* in thalassohaline hypersaline sites is enabled by both the success of Hqrw1 in high but stable salinities and the larger resistance of Hqrw2 to extreme osmotic stress, safeguarding the presence of the species in the system.

## Introduction

Hypersaline environments are globally distributed and constitute ideal systems to study microbial diversity, community structure and the effect of environmental disturbances due to their low complexity [1–3]. Among the key biological components of these systems, the species *Haloquadratum walsbyi* is probably the most successful inhabitant of hypersaline sites. This species generally dominates and can even represent up to ~80% of the total community (10^7^ cells/ml) in hypersaline environments [4–13]. The ecological relevance of *Hqr. walsbyi* in hypersaline systems could be comparable to the widely distributed and ecologically success of *“Pelagibacter ubique”* in the open oceans [14].

*Hqr. walsbyi* seems to be resistant to cultivation as yet less than ten isolates have been brought to pure culture, and only two genome sequences have been published [9, 15]. Comparative genomics of these two strains, C23^T^ and DSM 16790, isolated from two geographically distant hypersaline environments (Santa Pola in Spain and Lake Tyrrell in Australia, respectively, separated by ~17 000 km), revealed high genomic similarity, sharing 98.25% Average Nucleotide Identity [ANI] and minimal genomic rearrangements, with most variations being small insertions and deletions (4–20 bp) rather than large genomic islands [16]. This high genomic relatedness, alongside with low 16S rRNA gene sequence divergence (only two nucleotide differences) [15], led to the hypothesis that *Hqr. walsbyi* exhibits global homogeneity, potentially driven by high dispersal capability and strong evolutionary constraints, limiting allopatric differentiation [12]. Although an alternative hypothesis has been also proposed to explain similar global homogeneity in the sulfate reducer *Candidatus* Desulforudis audaxviator based on high-fidelity DNA replication and repair mechanisms, rather than fast and effective dispersal [17], the global homogeneity of *Hqr. walsbyi* via high dispersal capability has only been suggested based on two isolates [16] and 16S rRNA gene studies [12].

Subsequent population-level studies challenged this view of high homogeneity in *Hqr. walsbyi* by identifying multiple coexisting *Haloquadratum* clones, with some sharing one of the at least four genomic islands detected that may sum to a size of almost an additional chromosome (~3 Mb) in length, and being enriched with genes associated with mobile elements, cell envelope glycoproteins, and systems for detecting and transporting small molecules [18]. This accessory genome could enable substantial functional plasticity, promoting niche partitioning, ecological success under adverse conditions, and long-term persistence of the species in natural ecosystems [19–25]. However, a large auxiliary genome does not seem to be compatible with the high genomic relatedness observed for *Hqr. walsbyi* isolates. Consistent with our own previous study, we showed the coexistence of at least two highly related metagenome assembled genomes (MAGs), considered as different “ecotypes” as they exhibited clear niche partitioning, but maintaining high genomic relatedness [2]. One ecotype tended to dominate under stable, high-salt conditions, whereas another emerged and became equally abundant during periods of intermediate salinity (15%–23% salts). Despite their co-existence in local salterns (Mallorca, Spain) based on our previous study [2], the global distribution of these *Hqr. walsbyi* genomovars has not been evaluated.

Robust marine communities typically consist of a few dominant taxa along with numerous low-abundance populations [26–32]. Predominant taxa typically show better ecological success under non-dramatic environmental fluctuations (e.g. daily variation of light or temperature intensity) [19, 33–39]. Similar to these species-level patterns of dominance, several studies have reported that within a species only few coexisting subpopulations dominate, presumably due to better ecological fitness under stable conditions [25, 40–44]. We will refer to genomovars (i.e. groups of strains sharing ≥99.5% ANI and > 99.0% of their genes) [25, 45] as the basic units of diversity within a species as they reflect better genotypic and ecological consistency than strains according to our recent study [25], and as genomovars often show niche partitioning [25]. Actually, the two ecotypes of *Hqr. walsbyi* detected in the past [2] now would be grouped into distinct genomovars. Consistently, we have shown that the most abundant bacterial species in the hypersaline environments, *Salinibacter ruber*, consists of hundreds, if not thousands, genomovars that typically coexist, but only a few of them are abundant at each time point sampled. In contrast to *Hqr. walsbyi, the* intraspecific diversity of *Sal. ruber* is much larger, with ANI values averaging around 97.5% and ranging from 96.5% to 100% [24]. We also estimated that a single environment may contain 5000 to 11 000 genomovars, similar to what has been calculated for a single drop of seawater that can harbor hundreds of distinct strains [46–48]. Therefore, it remains unclear whether *Hqr. walsbyi* populations are also comprised of a high number of genomovars like *Sal. ruber*.

Alongside environmental filtering, which ultimately selects for the best-adapted species or genomovars [1, 2, 23–25, 36, 38], microbial communities thriving in hypersaline ecosystems are also generally controlled by high concentrations of viruses, typically representing up to 10^10^ viral-like particles (VLPs) per milliliter, among the largest reported for any aquatic ecosystem [49]. Furthermore, under environmental pressures, such as UV or dilution events, it has been demonstrated that viruses associated with *Hqr. walsbyi* increase in abundance, suggesting active infections after environmental disturbances [38, 50]. These viral-host interactions can be highly specific, with some viruses capable of infecting and exerting predation pressure on individual strains within a single species [51, 52]. However, due to the recent description of genomovars as a distinct unit within species [25, 45], phage-host interactions and dynamics at genomovar scale remain entirely unexplored. As a result, it is still unknown whether genomovars are targeted by unique viral species or by diverse consortia of viruses. Furthermore, it remains an open question whether such genomovar-associated viruses are endemic to particular locations or are globally distributed across hypersaline ecosystems.

In the present study, we focused on the intraspecies dynamics of *Hqr. walsbyi* and their viruses using the MAGs and viral OTUs (vOTUs) obtained from 130 metagenomes generated as part of our previous mesocosm study [38]. Additionally, we contrasted the results from the mesocosms with a global collection of MAGs and vOTUs derived from 24 metagenomes from 19 hypersaline environments across the world in an attempt to better understand the global dominance of this species and its unusually low intraspecific genome diversity.

## Materials and methods

### Mesocosm experiment on recurrent dilutions with variable intensity

**Figure 1:**
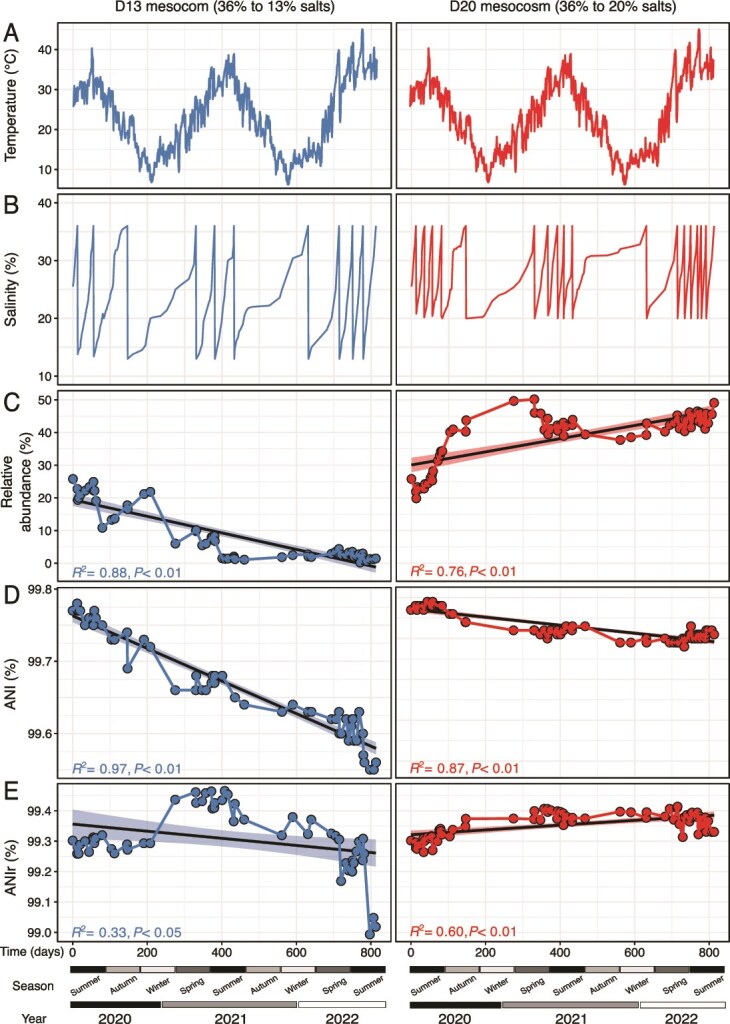
Influence of recurrent disturbances on *Hqr. walsbyi* abundance and intraspecies diversity. Oscillations in (A) temperature (°C) and (B) salinity (%) over the 813 days of mesocosm operation (modified from [38]). Temporal dynamics of *Hqr. walsbyi* are shown for (C) relative abundance, (D) genomic similarity (ANI), and (E) intraspecies diversity (ANIr), including trend lines over time with 95% confidence intervals (CIs), for the D13 (36% to 13% salts) and D20 (36% to 20% salts) mesocosms.

Data used here was obtained from two adjacent mesocosms monitored from June 10^th^ 2020 to September 1^st^ 2022 (813 days) that were established to understand how extreme halophilic microbial communities react to recurrent disturbances (Fig. 1) [38]. In brief, 640 L of brines collected from S’Avall solar saltern located in Mallorca (39°19′28″N; 2°59′21′′E) were divided into two ponds of 320 L each, and allowed to evaporate until salt saturation (>36% NaCl at 25°C; Fig. 1) [38]. Both ponds were subjected to a recurrent dilution treatment, one was diluted from 36% salts to 13% salinity (D13 mesocosm) and the other just to 20% salts (D20 mesocosm) by adding tap water flowing at a rate of 25 L/minute with continuous mixing to avoid stratification [38]. After dilution, the ponds were left to evaporate by natural sunlight until salt saturation was reached again, and a new round of dilution was initiated shortly after (Fig. 1) [38]. The influence of the tap water on the chemical and microbiological composition of the brine was negligible as reported previously [38]. Salinity and temperatures were monitored using a Sper Scientific Salt Refractometer and a HOBO Water Temp Pro v2 devices, respectively [38]. As previously discussed [38], biological replicates are not required for time-series experiments where microbial community variation remains minimal throughout the study period. Moreover, given the large experimental volume (i.e. 640 L) and the frequent sampling schedule (approximately every 2 weeks), performing replicates would be prohibitively expensive [38].

### Halophile sequencing project

**Figure 2:**
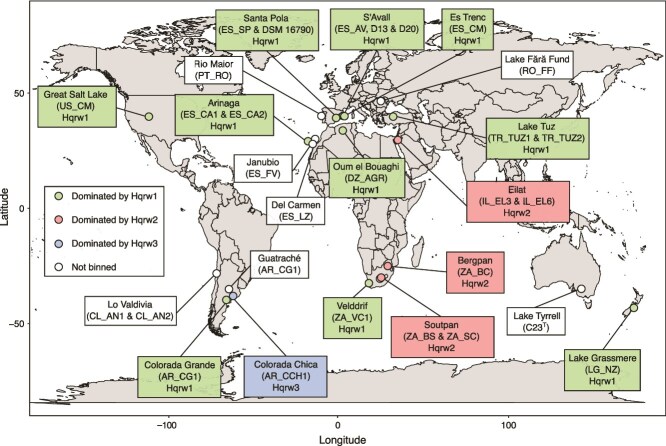
Location of the hypersaline sites sampled by the HSP. A map showing the HSP sampling sites used to estimate the global diversity of *Hqr. walsbyi*. Gradient indicates either which genomovar dominate each HSP sample or where *Hqr. walsbyi* did not bin.

To expand our understanding of the global distribution and composition of extreme halophilic microbial communities, an international consortium of scientists, the Halophile Sequencing Project (HSP), was formed in 2019 [53] (Supp. Fig. S1). The HSP consortium collected 24 hypersaline environments from 11 countries around the world with the aim to assess biogeographical patterns. Specifically, HSP samples represent both planktonic and viral communities collected from brines in the middle of the salterns at >30% salt concentration. Here, we used the 24 cellular metagenomes from HSP: S’Avall and Es Trenc, in Mallorca, Spain; Santa Pola in Alicante, and Arinaga, Janubio, and Del Carmen in the Canary Islands, both also in Spain; Eilat in Israel; Bergpan, Soutpan, and Velddrif in South Africa; Grassmere in New Zealand, Oum El Bouaghi-Sebkha Ezzemoul in Algeria; Tuz Lake in Turkey; Lo Valdivia in Chile; Great Salt Lake in USA; Colorada Grande, Colorada Chica, and Guatraché in Argentina; Rio Maior in Portugal, and *Fără* Fund in Romania (Fig. 2; Supp. Table S1).

### Sequencing and metagenomic analyses

Mesocosm metagenomes (59 from D13, and 70 from D20) were sequenced on a HiSeq System (Illumina; 2 x 150 bp, paired-end reads) at Macrogen, South Korea and HSP metagenomes (27) were sequenced on a NextSeq500 System (Illumina; 2 x 150 bp, paired-end reads) at Fisabio, Spain. Both metagenomic datasets were identically processed. Raw reads were trimmed using bbduk v38.82 (https://sourceforge.net/projects/bbmap/) with the following criteria: quality score ≥ 20 and length ≥ 100 bp. Trimmed reads were assembled using metaSPAdes v3.14.1 [54] retaining only contigs with a length ≥ 500 bp for subsequent analyses. Binning was performed with MetaBAT2 [55] and MaxBin v2.1.1 [56], using contigs of ≥2000 bp. Completeness and contamination were assessed using CheckM2 v0.1.3 [57], and only MAGs with an estimated completeness ≥50% and contamination <10% were included in further analyses. MAGs assigned to *Hqr. walsbyi* were identified using GTDB-Tk v2.1.1 [58] with the release207_v2 database. For each metagenome, the highest-quality MAG, determined by quality score (completeness −5 × contamination), was used for subsequent analyses.

### *In situ* species, genomovar, and gene abundances

Total species abundance was estimated by recruiting metagenomic reads of each sample against the MAG derived from each of these metagenomes. Following the same rationale as previously reported [25], genomovar abundances were calculated using the representative (i.e. highest quality) MAG of each genomovar as the reference genome in the read recruitment, and the relative abundance of genes was estimated using mapped reads against the representative sequence of each Orthology Group (OG) across metagenomes with Bowtie2 v2.3.4.1 [59] using the following parameters: –reorder –no-unal –f. The sequencing depth was estimated at each nucleotide position across the reference sequence by mapped reads using samtools v.1.10 [60] and bedtools [61], filtered at ≥95% identity for species abundance and at ≥99.3% for genomovar abundance as suggested previously [25]. The sequencing depth was further adjusted to represent only the middle 80% values (i.e. removing the upper and bottom 10% positions by depth) using *BedGraph.tad.rb* [62], providing the final TAD80 sequencing depth that was used as proxy for relative abundance. Because low-coverage regions can bias intraspecies diversity studies [63], MAGs with an estimated TAD80 sequencing depth below 10X were discarded for further analyses. Gene abundances across metagenomes were divided by the sequencing depth of *Hqr. walsbyi* obtained in the same metagenome to provide an estimate of copy number per genome [24].

### Intraspecific sequence and gene diversity analyses

ANI among genomes or MAGs, and intraspecific sequence diversity based on mapped reads against a reference genome sequence (i.e. ANIr) were calculated using the *ani.rb* and *anir.rb* scripts from the enveomics collection [62], respectively. Prodigal v2.6.3 [64] was used to predict genes and proteins from genomes and MAGs using default parameters. Identified genes in nucleotides were subsequently clustered into OGs using CD-HIT v4.8.1 [65] with parameters: –c 0.9 –n 8 –G 0 –g 1 –aS 0.7 –M 10000 –d 0 –T 10, selecting the largest gene as the representative of every OG. We grouped the OGs according to their occurrence using the parameter p = n/N, where n is the number of genomes or MAGs harboring a gene and N is the total number of genomes or MAGs analyzed. Specifically, OGs identified as core genes corresponded to p ≥ 90%, common to 20% ≤ p < 90%, rare when 1/N < p < 20%, or specific when p = 1/N, as suggested previously [24]. Functional annotation of OGs was performed using a custom script (https://github.com/rotheconrad/00_Annotation_Pipeline) with the TrEMBL database. Only OGs with ≥50% identity and ≥50% alignment with the TrEMBL database were kept for further metabolic comparisons among genomovars and locations.

### Viral contig identification, taxonomic classification, and clustering into viral OTUs

To ensure accurate identification, taxonomic classification and prediction of lytic and temperate viruses, contigs ≥5000 bp were extracted from metagenome assemblies and subjected to geNomad v.1.5.0 [66] with the following parameters: end-to-end —conservative. The identified viral contigs were clustered in vOTUs at ≥95% ANI with at least ≥85% coverage [67] relative to the shortest contig using CD-HIT v4.8.1 [65] (parameters: –c 0.95 –aS 0.85), selecting the longest sequence as representative of a given vOTU in subsequent analyses.

### Relative abundances of *Hqr. walsbyi* and vOTUs

Relative abundances were estimated using the representative sequence of each vOTU across metagenomes. For this, the trimmed reads were mapped to contigs using Bowtie2 v2.3.4.1 [59] and best-match mapped reads were filtered at 95% identity with samtools v.1.10 [60] and bedtools [61], and used to calculate the TAD80 sequencing depth as described above. To compare microbial and viral species abundances across metagenomes, sequencing depth (TAD80 values) was divided by the total number of reads in the metagenome and multiplied by 10^8^ (the sequencing effort).

### Viral-host assignation, and clustering in viral cohorts

Species virus-host linkage among vOTUs and *Hqr. walsbyi* was predicted using iPHoP v1.3.0 [68] with default parameters and score ≥ 75 [69]. The viral-host assignments at the species level were carried out selecting the putative host (including MAGs recovered from our dataset and available genomes from NCBI) with the highest iPHoP score obtained. As previously reported [38, 70], time-dependent correlations were used for identifying common patterns among viruses and hosts. Specifically, phage-genomovar correlations among vOTUs and their host (genomovars) were identified using the extended Local Similarity Analysis (eLSA) [71] for network analysis. Theoretical approximation (−p theo) was applied to estimate p-values, and only strong (*LS* score ≥ 0.6 or ≤ −0.6) and statistically significant (*P* ≤ 0.05; *Q* ≤ 0.01) correlations were retained [38, 70]. Viral clustering in “*viral cohorts*” and genomovar assignation to each viral cohort was performed using the Markov Clustering Algorithm (MCL) based on *LS* values obtained using eLSA approach [38, 70]. Viral gene functional predictions was performed as described above for *Hqr. walsbyi* genes.

### Ionic composition of HSP

HSP brines were diluted 1:400, filtered through 0.22 μm hydrophilic PTFE filters, and the filtrate was sent to the Technical Research Services of Alicante University, Spain for ion chromatography as previously described [13, 38, 53].

### Bioinformatic pipeline summary and statistical analyses

Materials and methods used in this study, including metagenome processing, binning, viral contig identification, clustering of vOTUs, genomovar identification, and phage-host linkages at species and genomovar levels, are summarized in Supp. Fig. S2. Non-metric multidimensional scaling (NMDS) based on ionic composition of HSP samples was conducted in R v4.1.2, using the vegan package. Statistical differences among datasets were determined using the Wilcoxon rank-sum test for pairwise comparisons and P-values were adjusted using the Benjamini–Hochberg correction. Significant differences were considered when *P-adjusted* < 0.05.

## Results

### *Hqr. walsbyi* intraspecies diversity in the mesocosms (local scale)

Distinct intensities of disturbance in the mesocosms had contrasting impacts on the whole community structures [38], benefiting the already dominant extreme halophiles in D20 (*Hqr. walsbyi* and *Sal. ruber*), and promoting the replacement of these dominant taxa by congeneric species under intense disturbances in D13 [38], consistent with our expectation that the 13% dilution represented a higher stress for these salt-adapted microbial communities [38].

From the 129 metagenomes collected during the 813 days of experimentation, 126 *Hqr. walsbyi* MAGs were binned (Supp. Table S1). Of these, 3 MAGs from the D13 mesocosm were excluded due to their sequencing depth being below 10X, which usually results in highly incomplete and/or contaminated MAGs (Supp. Table S1). Thus, the final dataset (n = 123) consisted of one MAG from time zero (12 June 2020; original brines), and 52 and 70 MAGs from the D13 and D20 mesocosms (22 June 2020 to 1 September 2022), respectively (Supp. Table S1). The average MAG length was 2.5 ± 0.4 Mbps, composed of 353 ± 82 contigs, with an average sequencing depth of 234X ± 169X (Supp. Table S1). Genome completeness averaged 84% ± 7.6%, with contamination averaging 0.8% ± 0.8% (Supp. Table S1). Mesocosm MAGs averaged 99.7% ± 0.1% ANI and 84.1% ± 5.3% of shared genome, with higher ANI values among D20 genomes (99.8% ± 0.1%) than among D13 genomes (99.7% ± 0.1%) (*P* < .05; Supp. Fig. S3).

Initially, *Hqr. walsbyi* was the most abundant species, representing 25.8% of the total community (total reads mapped; Fig. 1C). Before the first dilution, MAGs from both mesocosms shared 99.8% ANI, consistent with our expectation of high similarity of the *Hqr. walsbyi* populations in the two inocula used (Supp. Table S2). The moderate salinity disturbances in D20 led to a gradual increase in *Hqr. walsbyi* abundance from 25.8% to 49.1% by the end of the experiment (Fig. 1C), accompanied with only a slight decrease in ANI values, by 0.1% (*R*^2^ = 0.9; *P* < .01; Fig. 1D). In contrast, the higher intensity disturbance in D13 caused a marked decrease in *Hqr. walsbyi* abundance to ~1.5% of the total community, accompanied by a significant decline in ANI, by 0.2% by the end of the experiment (*R*^2^ = 0.9; *P* < .01; Fig. 1D).

### *Hqr. walsbyi* diversity in HSP samples (global scale)

To further evaluate the global diversity of *Hqr. walsbyi* we used the MAGs extracted from 17 out of 24 hypersaline sites of the HSP, where this species could be binned from (Fig. 2; Supp. Table S1). Specifically, *Hqr. walsbyi* MAGs were recovered from the hypersaline environments located in Algeria (Oum El Bouaghi-Sebkha Ezzemoul), Argentina (Colorada Grande and Colorada Chica), Israel (Eilat), New Zealand (Lake Grassmere), South Africa (Bergpan, Soutpan, and Velddrif), Spain (Arinaga, Es Trenc, S’Avall, and Santa Pola), Turkey (Lake Tuz), and USA (Great Salt Lake) (Fig. 2; Supp. Table S1). In the 17 hypersaline environments where the species could be binned, *Hqr. walsbyi* showed relative abundances ranging from 7.7% to 57.4% of the total microbial population based on read mapping, being particularly abundant in salterns from Spain, Algeria, Argentina, and New Zealand (Supp. Fig. S4). Among these, *Hqr. walsbyi* was the most abundant taxon in 13 out of 17 HSP samples. In the remaining four HSP samples, *Hqr. walsbyi* was surpassed in abundance by *Sal. ruber* and/or uncultured species of *Halobellus*, *Halonotius*, *Halorubrum*, and *Haloquadratum* (Supp. Fig. S4). Conversely, we were not able to bin *Hqr. walsbyi* (nor recruit enough reads against reference isolate genome) from the samples of Guatraché in Argentina, Lo Valdivia in Chile, Rio Maior in Portugal, *Fără* Fund in Romania, and both Janubio and Del Carmen in the Canary Islands in Spain (Fig. 2; Supp. Fig. S4). We thus concluded that *Hqr. walsbyi*, if present, was below the threshold of detection of our sequencing effort. The genomes of the only two cultivated isolates available were also included in our analysis: the strain DSM 16790 isolated from Santa Pola saltern in Alicante, Spain, and the type strain of the species C23^T^ isolated from Lake Tyrrell in Australia (Fig. 2). This resulted in a total of 19 genomes, which represented the global *Hqr. walsbyi* genome diversity (Fig. 2).

HSP MAGs had an average length of 2.5 ± 0.6 Mbps, 303 ± 165 contigs, and an average sequencing depth of 407X ± 253X (Supp. Table S1). Completeness and contamination averaged 86% ± 7.4% and 1% ± 1.3%, respectively (Supp. Table S1). These genomes exhibited high relatedness, with a range of ANI values from 98.3% to 99.9% and an average ANI of 99.4% ± 0.3% and genome sharing of 82.5% ± 7.6%, even among genomes separated by over 19 000 km (Fig. 2; Supp. Fig. S3). Therefore, the global and the local MAG populations harbored comparable levels of genome diversity overall.

### *Hqr. walsbyi* comprises four genomovars, with a single globally dominant genomovar

**Figure 3:**
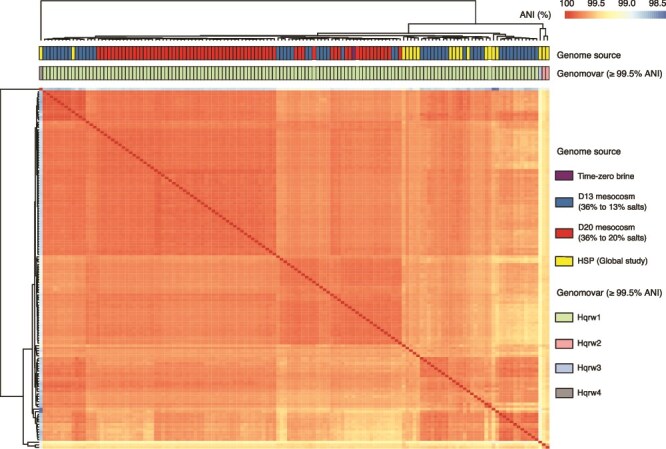
Genomic diversity of *Hqr. walsbyi* genomes. Heatmap showing the 142 x 142 ANI comparisons among D13 (36% to 13% salts), D20 (36% to 20% salts), and HSP (global study) *Hqr. walsbyi* genomes. Hierarchical clustering was conducted using the “average” algorithm with Euclidean distances. Genomovars were identified based on ANI values (see figure key for details).

ANI among all 142 genomes (i.e. 140 MAGs and two from isolates) revealed four distinct genomovars, with >99.5% ANI within a genomovar vs. *≤* 99.5% ANI between genomovars (Fig. 3; Supp. Fig. S5). The most frequently detected genomovar (Hqrw1) comprised the genome of DSM 16790 (Alicante, 2007) and MAGs recovered from 14 of 17 HSP metagenomes from Algeria, Argentina (Colorada Grande), New Zealand, South Africa (Bergpan, Soutpan, and Velddrif), Spain (Santa Pola, Es Trenc, S’Avall, and Arinaga), Turkey, and US (Supp. Table S1). It also encompassed the 123 *Hqr. walsbyi* MAGs recovered from both mesocosms. Collectively, the Hqrw1 MAGs clustered together with ANI values averaging 99.7% ± 0.1% and sharing 85.8% ± 5.7% of their genes (Fig. 3). The second genomovar (Hqrw2) consisted only of MAGs retrieved from Eilat salterns (Supp. Table S1), sharing 99.6% ANI and 85% of their genomes. The two most divergent genomovars, Hqrw3 (AR_CCH1 a single MAG from Colorada Chica, Argentina) and Hqrw4 (C23^T^ the single genome of the isolate from Lake Tyrrell, Australia, 2007), were represented by only one MAG or genome, respectively.

Hqrw1 showed an average ANI of 99.3% ± 0.1% and shared genome of 84.1% ± 4.9% with Hqrw2; an average ANI of 99.1% ± 0.1% and shared genome of 75.4% ± 4.6% with Hqrw3 and ANI of 98.7% ± 0.1% and shared genome of 86.3% ± 5.6% with Hqrw4 (Fig. 2; Supp. Table S1). Hqrw2, only binned in from Eilat, showed an average ANI of 99.2% ± 0.1% and shared genome of 70.5% ± 3.2% with Hqrw3 and an average ANI of 98.9% ± 0.2% and shared genome of 90.3% ± 2.8% with Hqrw4 (Fig. 2; Supp. Table S1). Hqrw3 and Hqrw4 showed an ANI of 98.9% and shared genome of 80.3% among them (Fig. 2; Supp. Table S1).

**Figure 4:**
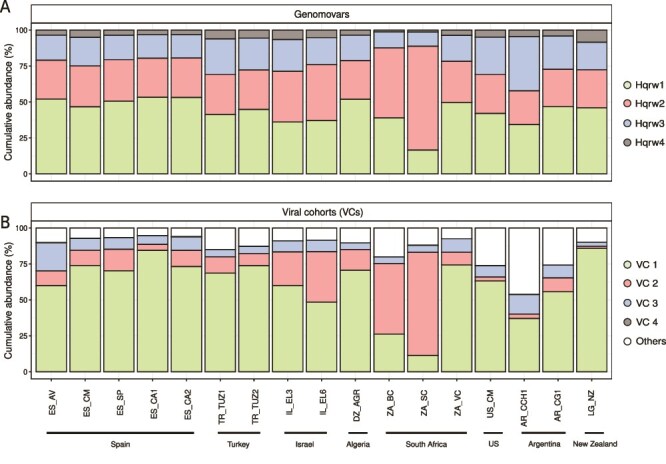
Genomovar and viral cohort abundances of *Hqr. walsbyi* at global scale. Barplot representing the cumulative abundance of (A) each genomovar and (B) their assigned viral cohorts across HSP (global study) metagenomes.

Competitive recruitment of metagenomic reads at high identity (i.e. ≥ 99.3%; or, allowing up to one mismatch per 150 bps read fragment), using the representative genome of each genomovar, was used to further understand the relative abundance of each *Hqr. walsbyi* genomovar in each HSP metagenome (Fig. 4A). All four genomovars were recruited in each of the 17 HSP samples where we obtained a *Hqr. walsbyi* MAG, which indicated co-occurrence of all genomovars globally. On average, these *Hqr. walsbyi* genomovars recruited 74.9% ± 5.1% of the total species abundance, ranging from 69.1% in South Africa (Soutpan) to 81.7% in Algeria (Oum El Bouaghi-Sebkha Ezzemoul). This competitive metagenomic read recruitment also revealed the dominance of Hqrw1 in 12 of 17 HSP samples analyzed (Fig. 4A). Among the total species abundance, Hqrw1 exhibited an average abundance of 31.7% ± 6.1% across these 17 sites, being particularly dominant in Algeria, Argentina (Colorada Grande), New Zealand, Spain, South Africa (Veldriff), Turkey, and US (Fig. 4A). The second most abundant genomovar was Hqrw2 representing 24.6% ± 6.7% of the total species abundance and dominating in both Eilat salterns and two South Africa salterns (Bergpan and Soutpan) (Fig. 4A). Genomovar Hqrw3 showed a global average relative abundance of 17.3% ± 5.6%, only being particularly abundant in Colorada Chica lagoon, comprising 33.7% of *Hqr. walsbyi* abundance in this lake (Fig. 4A). Finally, Hqrw4, being represented by the single isolate and type strain of the species C23^T^, exhibited the lowest relative abundance in all sites, averaging 4.3% ± 1.6% globally (Fig. 4A).

Consistent with the observed genomovar abundances in the HSP metagenomes (Fig. 4A), the average nucleotide identity of reads mapping to a MAG (ANIr), which is our preferred microdiversity metric due to its minimal sensitivity to different sequencing depth levels between metagenomes [63], indicated that the most divergent population compositions were observed in Colorada Chica (99.1% ANIr), Eilat salterns (~99.2% ANIr), and Soutpan (99.2% ANIr) (Supp. Fig. S6), aligning with a lower abundance of Hqrw1 and the higher relative abundance of Hqrw2 or Hqrw4 at these sites (Fig. 4A). In contrast, environments dominated by Hqrw1 exhibited lower intraspecies diversity, with an average ANIr of 99.4% ± 0.1% (Supp. Fig. S6).

### Genomovars and sites carry distinct content of transporter, membrane maintenance and mobile genes

HSP samples were collected from thalassohaline ecosystems dominated by sodium and chloride ions, with distinct proportions of manganese, sulfate, potassium, and bromide (Supp. Table S1; Supp. Fig. S7). Differences in ionic composition were observed between geographically close salterns such as between S’Avall and Es Trenc in Mallorca, between the two sampled ponds in Eilat, and the two close lakes in Argentina (Supp. Table S1; Supp. Fig. S7). The largest ionic composition difference was detected in the sample of Soutpan (ZA_SC) exhibiting the lowest salinity (25.8%) and the lowest concentrations of manganese (2 g/L) and potassium (0.4 g/L) (Supp. Table S1; Supp. Fig. S7). In contrast, Santa Pola (ES_SP) showed the highest salinity (40.8%). Differences in ionic composition were also observed in Bergpan (ZA_BC), with the highest bromide concentration (4 g/L), in the two Canary Islands salterns, both with salinities below 30%, in Colorada Chica with the highest concentration of phosphate (0.09 g/L), and in Eilat with the highest concentration of potassium (11.1 g/L) (Supp. Table S1; Supp. Fig. S7).

Given the observed differences in ionic composition among HSP samples, we aimed at evaluating whether the environmental conditions of the HSP selected for specific gene repertoires of the genomovars present in them. By functionally annotating the pangenome of the 142 MAGs and genomes we detected 10 859 non-redundant genes and an open pangenome (γ = 0.46; γ reflects the slope of the rarefaction curve representing total number of non-redundant genes), with 76.9% of the genes being genomovar-specific (Supp. Text S1; Supp. Fig. S8; Supp. Table S2). Most genomovar or site unique genes were largely hypothetical, along with several organic and inorganic solute transporters, genes related to membrane maintenance, mobile elements, and phage-resistance genes (Supp. Text S1; Supp. Fig. S9; Supp. Table S2).

### Hqrw2 shows more resistance under osmotic stress than the other genomovars

**Figure 5:**
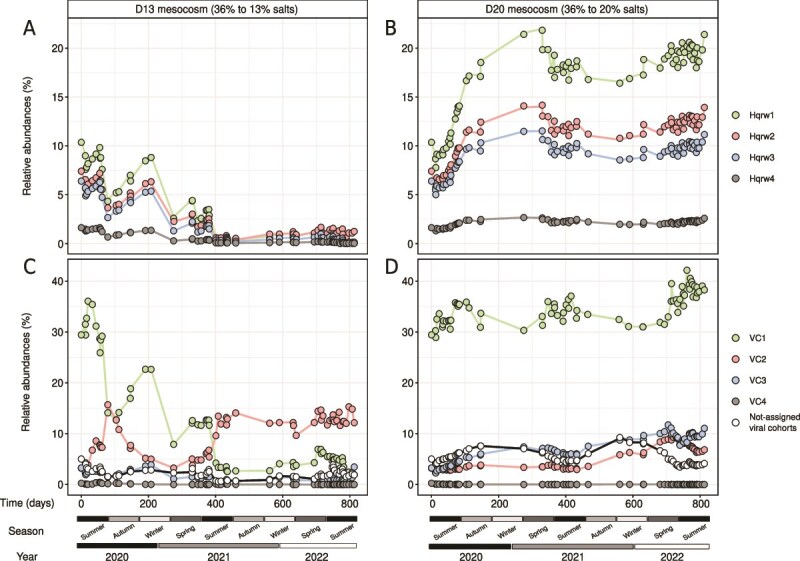
Genomovar and viral cohort dynamics of *Hqr. walsbyi* at mesocosm scale. Lineplots representing the relative abundance of each genomovar in (A) D13 (36% to 13% salts) and (B) D20 (36% to 20% salts) mesocosms. Lineplots representing the relative abundance of their assigned viral cohorts in (C) D13 (36% to 13% salts) and (D) D20 (36% to 20% salts) mesocosms.

As intense disturbances had pronounced impacts on both whole-community structure [38] and *Hqr. walsbyi* diversity (Fig. 1C and D), we evaluated the dynamics of genomovars in both mesocosms in detail (Fig. 5A). Competitive recruitment using high-identity reads revealed that the four *Hqr. walsbyi* genomovars recruited on average 75.5% ± 33% of total reads assigned to the species, ranging from 71% to 79.6% in the D13 mesocosm and 79.9% ± 0.9% in the D20 mesocosms (Supp. Figure S10). In D20, the percentage of reads mapping at 99.3% to an available *Hqr. walsbyi* MAG increased over time, whereas in D13, the percentage of reads strongly decreased from day 408 and did not recover along the following 405 days of experiment. Consistently, we also observed a steady increase of ANIr over time in D20, reflecting a decrease in intraspecies diversity (or more clonal population; *R*^2^ = 0.6; *P* < .01; Fig. 1E), and conversely, in D13 we observed a general decline in ANIr values, from 99.3% to 99.0% (*R*^2^ = 0.4; *P* < .05; Fig. 1E), suggesting the establishment of a more heterogeneous population.

Competitive recruitments also showed that the original brines were initially dominated by Hqrw1, with a relative abundance of 10.5%, followed by Hqrw2 (7.3%), Hqrw3 (6.4%), and Hqrw4 (1.6%) (Fig. 5A and B). In D20, we detected an approximately two-fold increase of Hqrw1 and Hqrw2, over time, followed by a 1.75-fold increase of Hqrw3 and a 1.5-fold increase of Hqrw4 (Fig. 5B). Conversely, in D13, the more intense disturbances led to a much more pronounced shift with 45- to 85-fold decreases observed for Hqrw1 (10.5% to 0.2%), Hqrw3 (6.4% to 0.1%), and Hqrw4 (1.6% to 0.1%) from time-zero to 813 days (Fig. 5A). Although Hqrw2 also decreased in overall abundance as consequence of the intense disturbances, the decrease was lower than the remaining genomovars (7.3% to 1.1%) (Fig. 5A) causing it to dominate the population at the end of the incubation by 5.5-fold over Hqrw1.

### Viral succession mirrors *Hqr. walsbyi* intraspecific shifts

A total of 802 and 38 056 viral contigs were assembled from the HSP and mesocosm experiments, respectively, for the global host microbial community (Supp. Table S3). 99.1% (n = 38 523) of these viral contigs were classified as lytic, whereas only 0.9% (n = 335) were assigned as prophages (Supp. Table S3). After dereplication at the species level (≥ 95% identity and ≥ 85% alignment), these 38 858 viral contigs were clustered into 8067 vOTUs (Supp. Table S4), which only 1.4% (n = 115) were shared between HSP and mesocosm metagenomes, whereas 98.6% were specific to either HSP (n = 590 vOTUs) or mesocosms (n = 7362 vOTUs) (Supp. Table S4). Read recruitment analysis revealed that 66.8% of these vOTUs (n = 5392) were present in at least one sample from both HSP (n = 17) and mesocosm (n = 130) datasets, whereas 2.8% (n = 224) were exclusive to HSP and 30.4% (n = 2451) were specific to the mesocosm experiments (Supp. Fig. S11; Supp. Table S4). Among these, 201 vOTUs were detected across all 147 samples from D13, D20, and HSP, despite originating from distant environments such as Argentina, New Zealand, Spain, and Turkey (Supp. Fig. S11; Supp. Table S4).

**Figure 6:**
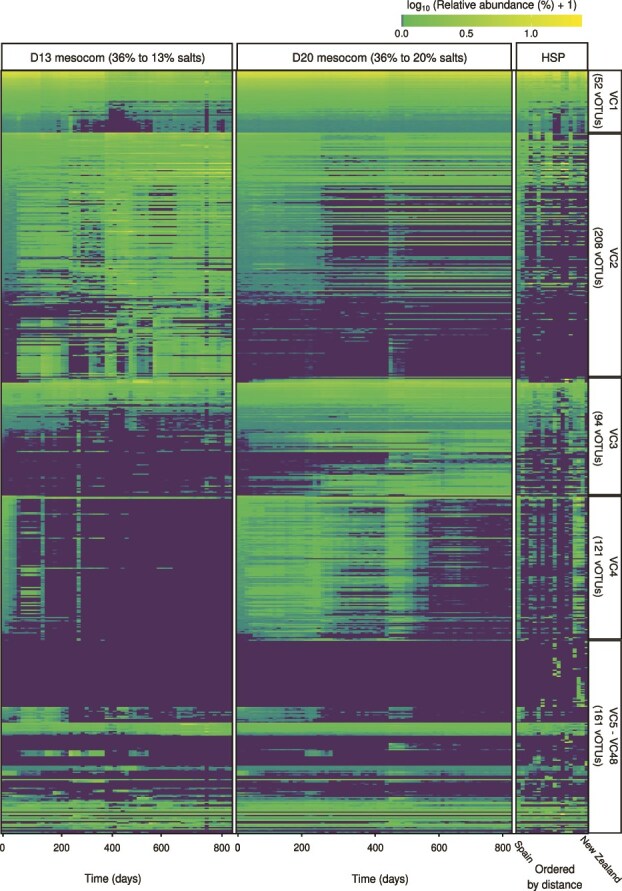
High heterogeneity of viral abundances at both mesocosm and global scales. Heatmap representing the relative abundance at logaritmic scale of each vOTU, grouped by viral cohorts, along the 813 days of experimentation in D13 (36% to 13% salts) and D20 (36% to 20% salts) metagenomes, and across HSP (global study) samples. Note that HSP samples were ordered by distance (km) from Spain to New Zealand hypersaline environments.

From the 8067 vOTUs detected, 636 were assigned to *Hqr. walsbyi* (iPHoP score > 75). However, we did not find exact matches (i.e. 100% identity) between viruses and genomovars in their CRISPR-based linkages (Fig. 6; Supp. Table S4). Furthermore, none of these vOTUs contained any identified auxiliary metabolic genes that could influence host dynamics by providing a metabolic advantages (Supp. Table S5).

Similar to the overall viral community, a substantial proportion of the vOTUs (n = 498; 78.3%) was shared between the two datasets, whereas 63 and 75 vOTUs were exclusive to the mesocosm and HSP datasets, respectively (Fig. 6; Supp. Table S4). These *Hqr. walsbyi* vOTUs also exhibited global distributions, with 82 vOTUs consistently detected across all D13, D20, and HSP samples (Fig. 6; Supp. Table S4). These *636* vOTUs accounted for 40% of the total viral community in the beginning of the experiment (Supp. Fig. S12). In D20, vOTUs increased significantly over time, reaching a relative abundance of 60.3% at 813 days (Supp. Fig. S12), whereas in D13, *these* declined to 25.5% after 813 days (Supp. Fig. S12), following the dynamics of their (predicted) host (Fig. 1C). Moreover, the relative abundances of vOTUs infecting *Hqr. walsbyi* in HSP collection showed an average of 53.3% ± 12.6%, ranging from ~31.8% in Arinaga (ES_CA1) to 74.8% in Tuz Lake (TR_TUZ1) (Supp. Fig. S13).

Despite putatively infecting the same host, *Hqr. walsbyi* vOTUs exhibited highly variable abundance patterns at both the local and global scales (Fig. 6; Supp. Table S6). Over the course of the 813-day experiment, 164 vOTUs (25.8%) remained consistently detectable (> 120 samples), whereas the majority (74.2%) fluctuated significantly, with 146 and 141 viruses emerging over time, 205 and 202 disappearing after initial detection, or 185 and 129 appearing sporadically along D13 and D20 mesocosms, respectively (Fig. 6; Supp. Table S6). At the global scale, some vOTUs were consistently abundant across samples, whereas others were only prevalent in particular samples, remaining absent in others (Fig. 6; Supp. Table S6).

Assuming that similar dynamics over time would indicate infection by the viral genomes of the same host population [68], we clustered *Hqr. walsbyi* vOTUs into “viral cohorts” [38]. We identified 48 distinct cohorts (Fig. 6; Supp. Table S6), 19 of which were represented by one vOTU, and the remaining 29 were composed of two or more vOTUs, with five cohorts comprising 83.3% (n = 530) of the 636 vOTUs predicted to infect *Hqr. walsbyi* (Fig. 6; Supp. Table S6). Only four viral cohorts were exclusive to the mesocosm experiments, whereas 15 were present along both mesocosm and HSP datasets (Fig. 6; Supp. Table S6). Among these, four cohorts were specifically associated with each of the four *Hqr. walsbyi* genomovars based on their common abundances (*LS* > 0.6; *P* < .01; Supp. Table S6). Specifically, VC1 (n = 52 vOTUs), VC2 (n = 208 vOTUs), VC3 (n = 94 vOTUs), and VC4 (n = 121 vOTUs) were predicted to infect Hqrw1, Hqrw2, Hqrw3, and Hqrw4, respectively (Fig. 6; Supp. Table S6). In D13, the dominant viral cohort (VC1 infecting Hqrw1) declined over time and this reduction was accompanied by an increase of the viral cohort VC2 (infecting Hqrw2; Fig. 5C), aligning with shifts in the dynamics of the corresponding genomovar host (Fig. 5A). In contrast, changes in the relative abundances of viral cohorts in D20 showed that moderate disturbances did not impact the dominance of VC1 (Fig. 5D). Despite this higher stability in D20, we found a clear decrease in abundance of VC1 during the second autumn-winter event (Fig. 5D). The very similar pattern dynamics between the genomovar and their assigned viral cohort may indicate that these viral populations may be specific to a single genomovar.

Analysis of the HSP metagenomes indicated a global dominance of VC1 infecting Hqrw1 (Fig. 4B), being the most abundant cohort in 14 hypersaline ecosystems. Consistently, we detected a higher relative abundance of VC2 Hqrw2 in Israel and South Africa (Bergpan and Soutpan) and large abundance of VC3 in Argentina (Colorada Chica) (Fig. 4B), which coincided with the higher abundance of Hqrw2 and Hqrw3 in these environments, respectively (Fig. 4A). Despite Hqrw4 being detectable in all HSP metagenomes (Fig. 4B), the relative abundances of its assigned viruses were minimal (< 0.5%; Fig. 4A).

## Discussion

### *Hqr. walsbyi* exhibits high intraspecies similarity

*Hqr. walsbyi* is the most abundant extreme halophile in most aerobic thalassohaline brines sampled globally [4–13], with few exceptions such as the previously studied Cáhuil system in Chile [72] or the Fără Fund (Romania), Del Carmen (Lanzarote Island, Spain), Rio Maior (Portugal), and Lo Valdivia (Chile) characterized by this study. Other salterns such as the Eilat (Israel) and South African salterns, *Hqr. walsbyi* was present but not dominant. *Hqr. walsbyi* exhibited low intraspecific diversity, with genomic relatedness >98.3% ANI among all MAGs and isolate genomes recovered from sites that are >19 000 km apart, as also observed for the only two isolates available, DSM 16790 and C23^T^ [16, 73, 74]. This high genomic homogeneity contrasts with the much greater diversity observed in other dominant and cosmopolitan bacterial species inhabiting marine, freshwater, or extreme ecosystems, such as *Polynucleobacter paneuropaeus* (average ANI within the species <97%) [39], *Prochlorococcus marinus* (< 97% ANI) [75], “*Pelagibacter ubique”* (93%–94% ANI) [14], *Sal. ruber* (~98% ANI) [24, 25]; and also the thermoacidophilic archaeon *Sulfolobus islandicus* (~98% ANI) [76] or the extremely halophilic archaeon *Halorubrum ezzemoulense* (<98.3% ANI) [77]. This high level of homogeneity is only comparable to that of *Cand.* Dsv. audaxviator (99.2% ANI) [17], in which its slow genome evolution was speculated to be the result from its extremely effective DNA replication and repair mechanisms [17].

Among all genomes retrieved, that of the type strain of *Hqr. walsbyi* C23^T^ [15] is the most divergent and probably the least representative of the species in terms of ecological distribution and/or gene content. Even though this does not affect the phylogenetic placement of the species and thus its classification, it is a good example that nomenclatural types based on isolates may not represent the taxon well, even in the case of a species with reduced diversity such as *Hqr. walsbyi*. The isolate DSM 16790, belonging to the globally dominant Hqrw1 that makes up the majority of the MAGs reported here, would be much better representative of the species in terms of functional gene content, genome sequence/alleles, and ecological distribution.

### Evidence for allopatric speciation in *Hqr. walsbyi*

As suggested previously for Ca. Dsv. Audaxviator [17], effective DNA replication and repair mechanisms could explain the intriguing global dominance of *Hqr. walsbyi* and the apparently low levels of allopatric speciation. However, we also suggest that these results may also be a reflection of a highly effective mechanism of dispersion and homogenization resulting from the globally circulating marine currents (mainly for the coastal sites), combined with aerial [78] or migrating bird-mediated dispersions [79]. In addition to the global dominance and low intraspecific diversity, we also detected that just four genomovars, that collectively make up 70 to 80% (or more) of the total species population, dominated in all sampled sites. Although the exact mechanism(s) causing the emergence of new genomovars remains unclear [25, 80], we show that each seems to have distinct ecological preferences and distinct associated functional gene content related to adaptation of location-specific ionic composition. Specifically, genomovar Hqrw1 was globally the most successful as it dominated almost all sites sampled, and one reason could be its apparent superior fitness under stable high-saline conditions as deduced from its increased dominance in the D20 experiment over time in which salinity shifts were small [38]. The reasons for its fitness could primarily be due to its large repertoire of transporters and membrane maintenance genes [2]. Many of the brines studied here originate from solar salterns where the human control of the evaporation-feeding cycles which provide a relatively stable environment in terms of osmotic changes that ultimately would select for Hqrw1 with the fittest phenotype [2, 38, 81]. However, the same genomovar Hqrw1 also dominated in “naturally” occurring brines like Colorada Grande Lagoon, Lakes Grassmere and Tuz and the Great Salt Lake, indicating that human activities are not the only or even main selection pressure.

### Local environmental conditions presumably shape the diversity of *Hqr. walsbyi*

In contrast to the global dominance of Hqrw1, we observed that other genomovars may dominate some inland hypersaline environments like the solar salterns of Bergpan and Soutpan, the Colorada Chica lagoon, and also the coastal solar saltern of Eilat. This discordance may be due to either determined site-specific physicochemical characteristics (e.g. larger phosphate in Colorada Chica) or undetermined environmental fluctuations that may have preceded our single-date sampling. Intense and recurrent osmotic stresses below the 15% salt threshold strongly influence the microbial community structures [2, 38, 82], and such intense changes exerted here appear to disfavor Hqrw1 over Hqrw2, which seems capable of enduring a broader osmotic range. One of the reasons for a better fitness of Hqrw2 under a changing environment may be related to its gene content as its unique halomucin gene that has been described as essential for resisting osmotic changes and phage predation [83], or the better fitness of some of its genomovar-specific solute transporters as previously observed [2, 18]. Such genes are generally encoded in hypervariable genomic islands or plasmids potentially acquired through horizontal gene transfer [2, 18, 24, 84–86]. Although intense disturbances negatively impacted *Hqr. walsbyi* abundance by especially reducing the dominance of Hqrw1, the stronger resistance of Hqrw2 seem to prevent or mitigate the displacement of the species under changing environmental conditions. The distinct fitness among genomovars apparently allows the species as a whole to rapidly respond to and survive environmental stressors, enhancing the long-term stability and functional resilience of the community, as hypothesized previously based on modeling and/or ecological theory [87–89].

### Low intraspecies diversity within *Hqr. walsbyi* compared to other saltern taxa

Here we show a global prevalence of *Hqr. walsbyi* in hypersaline environments, with a low intraspecies diversity in where just a small number of genomovars coexist. This contrasts with our previously studies based on hundreds of isolates of *Sal. ruber* for which the over 100 genomovars detected. These genomovars were roughly equally *in situ* abundant leading to an estimated richness ranging from 5000 to 16 000 genomovars in a single site [25]. Projecting the same evaluation for *Hqr. walsbyi* it seems that at a single site richness would be ~30-fold less diverse. However, we are aware that the use of MAGs is not accurate enough for this type of analysis and, for example, single-cell sorting and Single-cell Amplified Genomes (SAGs) may help to solidly quantify the level richness observed for a species that is very recalcitrant to be isolated in pure culture. Nonetheless, the much lower intraspecies diversity of *Hqr. walsbyi* is remarkable compared to other species that are highly successful in salterns or other relevant species such as the *Escherichia coli*.

### Yet unknown cohesiveness mechanism(s) may promote large homogeneity within *Hqr. walsbyi*

Given that *Sal. ruber* (as well as archaeal species being prevalent at the same sites across the globe) is likely subjected to the same dispersion mechanisms and aerial transport as *Hqr. walsbyi*, the much lower level of intraspecific diversity observed in the latter vs. the former cannot solely attributed to efficient dispersal mechanisms. Other ecological and/or genetic mechanisms must be responsible for their striking difference in intraspecies diversity. These mechanisms could include homologous recombination, with the key hypothesis to be that recombination is much more frequent within *Hqr. walsbyi* than within *Sal. ruber* based on a few previous studies [17, 80]. Further, homologous recombination that has been shown to play a critical role in archaeal genome evolution, facilitating speciation, adaptation, and the maintenance of diversity [90–93]. Many of the horizontally acquired genes in Archaea seem to be involved in metabolism and cell envelope biogenesis, and probably provide a selective advantage in adaption to new ecological niches [94, 95]. These findings could also apply to *Hqr. walsbyi*, which has been described as highly recombinogenic [18, 73], with genomic islands enriched with genes associated with mobile elements, glycoproteins involved in the cell envelope, and systems for detecting and transporting inorganic and organic molecules [18], a fact that strongly aligns with the observed differences among *Hqr. walsbyi* genomovars by our study. Another plausible explanation for the lower genomic heterogeneity is the frequent occurrence of oligoploidy and polyploidy in most, if not all, haloarchaeal species [96–98] that can affect genome assembly and binning during metagenomic analyses, potentially leading to the random recovery of different replicons of the same species as of the best-quality MAGs [99, 100]. Although oligo- or polyploidy has not been experimentally confirmed in *Hqr. walsbyi* yet, both available genomes of this species contain multiple origins of replication [16, 101], consistent with a probable polyploid lifestyle. One potential consequence of polyploidy is enhanced DNA repair capacity, which can reduce mutation rates and promote genome stability [98, 99]. These factors may contribute to the features observed here, that should be experimentally tested in future studies combining single-cell genomics, cultures, and MAGs.

### Phage dynamics supports genomovar abundances at both local and global scales

Recent advances in viral identification [66], viral-host prediction [68], and time-dependent correlations [38, 70] have enabled the monitoring of specific viral-genomovar linkages within species over extensive time-series and across geographically distant hypersaline systems. These analyses reveal that shifts in genomovar abundances are closely tied to changes in their associated viral cohorts, emphasizing the ecological significance of virus-host interactions in extreme environments. The detection of more than 65% of vOTUs across both mesocosm and HSP datasets, even in geographically distant salterns such as Mallorca (Spain) and New Zealand (> 19 000 km apart), further supports the hypothesis of the global connectivity of microbial and their corresponding viral communities inhabiting hypersaline systems. This is reinforced by our results on the global occurrence and dominance of 82 vOTUs clustered into 15 viral cohorts. The detection of putative prophages viruses in all samples supports the dispersal-based connectivity models in viral ecology, which propose that viruses persist across distant ecosystems, potentially facilitated by atmospheric and oceanic currents, migratory vectors or inside the cells as temperate viruses [102–106].

Given that viruses seem to be the primary regulators of microbial communities in hypersaline environments [107], the worldwide presence of ~500 vOTUs predicted to infect *Hqr. walsbyi* underscores the global dominance of the species. The strong correlation between genomovars and their clearly assigned viral cohorts suggests a high host-specificity, and an active regulation of the abundance of their aligning with the classical kill-the-winner model [72, 108–112]. However, a substantial fraction of the vOTUs displayed non-correlated dynamics with the four dominant genomovars, that may indicate the presence of cryptic (undetected) genomovar diversity, which could emerge when environmental conditions become favorable, and, therefore, a proliferation of their viruses [26, 113]; or may suggest that these cohorts were not genomovar-specific, but of a wider host-range and low efficiency. Future studies with higher sampling frequency (i.e. minute- or hour-scale intervals) combined with ecological modeling would unveil the reasons.

Altogether, it seems that the extremely successful nature of *Hqr. walsbyi* may be directly related to the coexistence and genomic homogenization of a small number but very fit abundant genomovars, controlled by their specific viruses, that ultimately would prevent the species as a whole from being purged from hypersaline systems with high connectivity and very effective dispersal mechanisms. The striking observation of the extremely low genomic diversity and the single genomovar global domination suggests a potential for alternate modes of genetic and ecological differentiation*,* which should be subject of future research.

## Sampling permits

Brines collected from the Spanish Solar Salterns were authorized by the Spanish Ministry of Ecological Transition with the permit numbers: ESNC22 and ESNC27. Samples from Great Salt Lake were collected in accordance with permission from the State of Utah. BKB retains all necessary permits from the Utah Division of Forestry, Fire, and State Lands for sampling at Great Salt Lake. Landowner permissions were obtained for sampling of the South African salterns. Local security authorities allowed sampling at the Oum El Bouaghi-Sebkha Ezzemoul hypersaline system in Algeria. Samples from Turkey were collected in accordance with the permission from the salt production site operators of Tuz Lake. Samples from Argentina were collected under permit issued by the “*Dirección de Recursos Naturales”* of the Province of La Pampa (Argentina), operating under the Ministry of Production (“*Subsecretaría de Asuntos Agrarios”*). At the time, all required transport permits (“*guías”*) for the legal transfer of the samples were also obtained. All sampling campaigns were done in accordance with compliance with the permit requirements in each country and/or region.

## Supplementary Material

Bustos-Caparros_Supp_Material_Third_Resubmission_wraf165

Bustos-Caparros_Supp_Tables_Third_Resubmission_wraf165

## Data Availability

Datasets used in the current study are available in the European Nucleotide Archive (ENA) repository at https://www.ebi.ac.uk/ena/browser/home, under BioProject accession numbers PRJEB75750 and PRJEB45291. Pangenome analysis and statistics of *Hqr. walsbyi* MAGs and isolates were assessed by a custom pipeline (available at: https://github.com/rotheconrad/00_Pangenome_Analysis).
