# Supplementary material for: Global dominance of Haloquadratum walsbyi by a single genomovar with distinct gene content and viral cohorts from close relatives
Source: ISME J. 2025 Aug 4;19(1):wraf165. doi: 10.1093/ismejo/wraf165 (PMC12400929; doi:10.1093/ismejo/wraf165)

**Global dominance of *Haloquadratum walsbyi* by a single highly clonal genomovar with distinct gene content and viral cohorts from close relatives**

Esteban Bustos-Caparros^1^*, Tomeu Viver^1^, Juan F. Gago^1^, Juanita R. Avontuur^1^, Souad Amiour^2^, Bonnie K. Baxter^3^, María E. Llames^4^, Mehmet B. Mutlu^5^, Aharon Oren^6^, Ana S. Ramírez^7^, Matthew B. Stott^8^, Stephanus N. Venter^9^, Fernando Santos^10^, Josefa Antón^10^, Luis M. Rodriguez-R^11^, Rafael Bosch^12^, Brian P. Hedlund^13^, Konstantinos T. Konstantinidis^14^, Ramon Rossello-Mora^1^*.

^1^Marine Microbiology Group (MMG), Department of Animal and Microbial Biodiversity, Mediterranean Institute for Advanced Studies (IMEDEA, CSIC-UIB), Esporles, Spain.

^2^Laboratory of Bioengineering, National Higher School of Biotechnology, Constantine, Algeria.

^3^Great Salt Lake Institute, Westminster University, Salt Lake City, UT, 84105, USA.

^4^Laboratorio de Ecología Microbiana Ambiental, Instituto Tecnológico de Chascomús (CONICET-UNSAM), Escuela de Bio y Nanotecnologías (UNSAM), Buenos Aires, Argentina.

^5^Department of Biology, Eskisehir Technical University, Eskisehir, Turkey.

^6^Department of Plant and Environmental Sciences, The Institute of Life Sciences, The Hebrew University of Jerusalem, Edmond J. Safra Campus, Jerusalem, 9190401, Israel.

^7^Unidad de Epidemiología y Medicina Preventiva, IUSA, Facultad de Veterinaria, Universidad de Las Palmas de Gran Canaria, C/ Trasmontaña s/n, Arucas, 35413, Canary Islands, Spain.

^8^School of Biological Sciences, University of Canterbury, Christchurch, New Zealand.

^9^Department of Biochemistry, Genetics and Microbiology, and Forestry and Agricultural Biotechnology Institute (FABI), University of Pretoria, Pretoria, South Africa.

^10^Department of Physiology, Genetics and Microbiology, University of Alicante, 03690, San Vicent del Raspeig, Alicante, Spain.

^11^Department of Microbiology and Digital Science Center (DiSC), University of Innsbruck, Innsbruck, Austria.

^12^Microbiologia, Departament de Biologia, Edifici Guillem Colom, Universitat de les Illes Balears, Campus UIB, 07122 Palma de Mallorca, Spain.

^13^School of Life Sciences, University of Nevada, Las Vegas, NV 89154-4004, USA.

^14^School of Civil and Environmental Engineering, Georgia Institute of Technology, Atlanta, GA, USA.

*Corresponding authors: Esteban Bustos-Caparros, Marine Microbiology Group (MMG), Department of Animal and Microbial Biodiversity, Mediterranean Institute for Advanced Studies (IMEDEA, CSIC-UIB), C/ Miquel Marquès 21, Esporles, Illes Balears, 07190, Spain. Email: [ebustos@imedea.uib-csic.es](mailto:ebustos@imedea.uib-csic.es), Ramon Rossello-Mora, Marine Microbiology Group (MMG), Department of Animal and Microbial Biodiversity, Mediterranean Institute for Advanced Studies (IMEDEA, CSIC-UIB), C/ Miquel Marquès 21, Esporles, Illes Balears, 07190, Spain. Email: [ramon@imedea.uib-csic.es](mailto:ramon@imedea.uib-csic.es).

**Running title: Microdiversity drives species success**

**Supplementary Figure S1.** Location of the hypersaline sites sampled by the Halophile Sequencing Project (HSP).


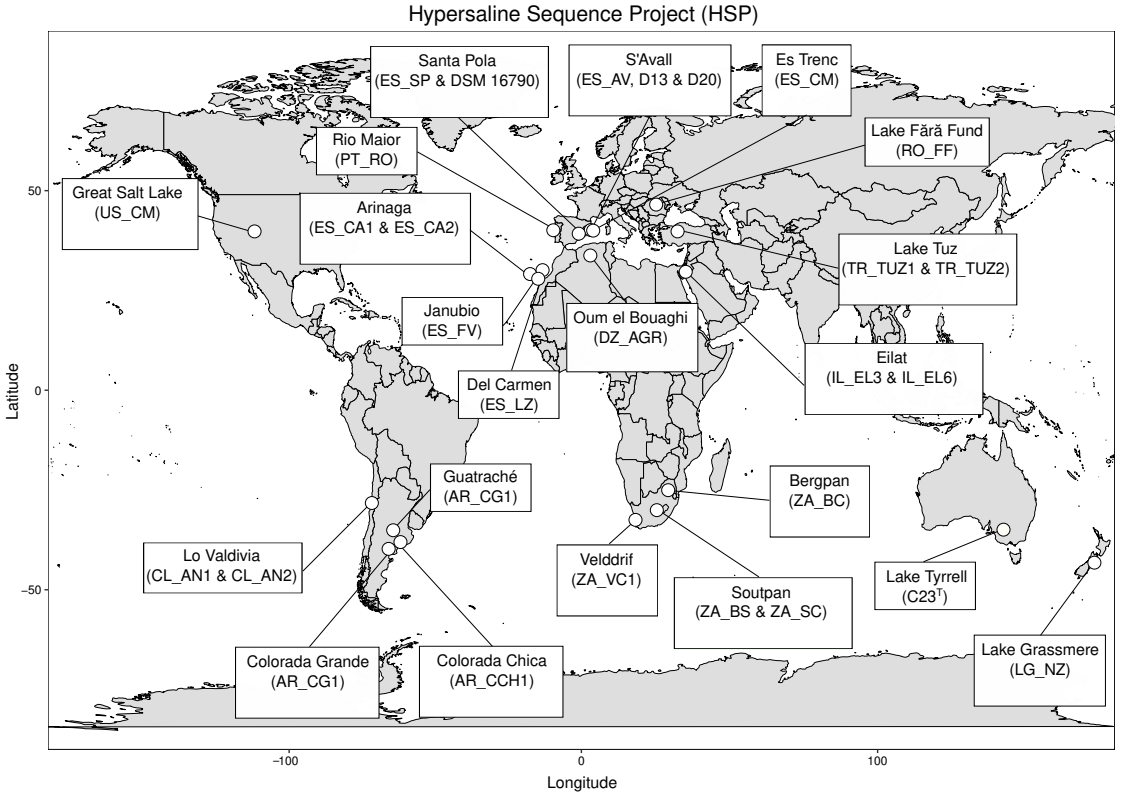


**Supplementary Figure S2.** Summary of the metagenomic procedure followed in this study, including trimming, assembly, binning, viral contig identification, clustering in species and vOTUs, and phage-host assignations at species and genomovar levels.


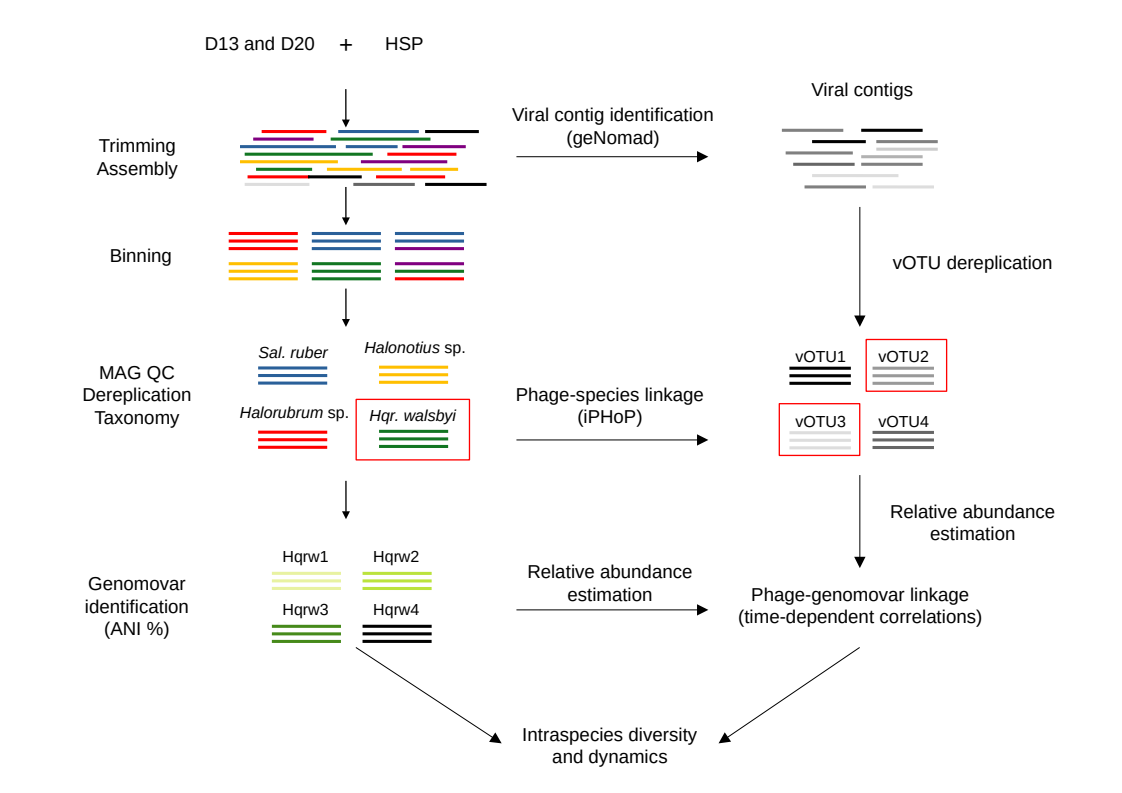


**Supplementary Figure S3:** Combined boxplots and violinplots representing ANI (%) across D13, D20, and HSP genomes. Statistical differences were determined using the Wilcoxon rank-sum test for pairwise comparisons and P-values were adjusted using the Benjamini-Hochberg (BH) correction, and significant differences are indicated with ** (*P* < 0.01).


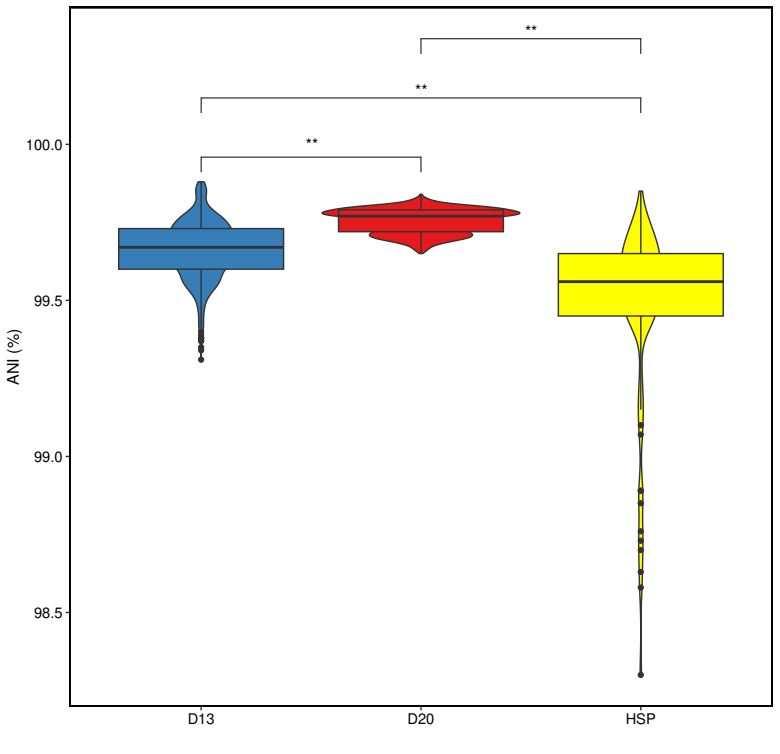


**Supplementary Figure S4:** Relative abundances, based on read mapping, of the nine most abundant species within the HSP project.


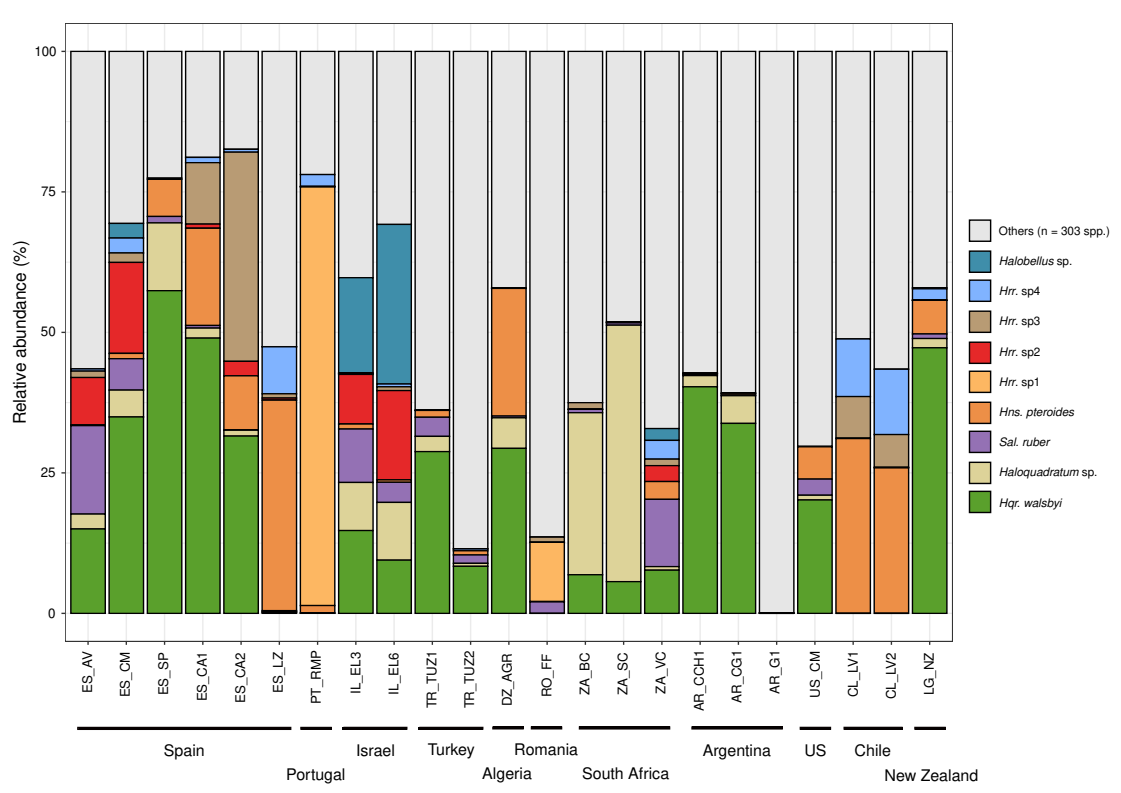


**Supplementary Figure S5:** The central panel represents the comparison between Hqrw1 genomes against Hqrw2, Hqrw3, and Hqrw4 showing their ANI value (x-axis) and the shared genome percentage (y-axis). The marginal plots represent the distribution of comparison for ANI value (x-axis) and the shared genome percentage (y-axis), which distinguish between the four-genomovar collection.

​​
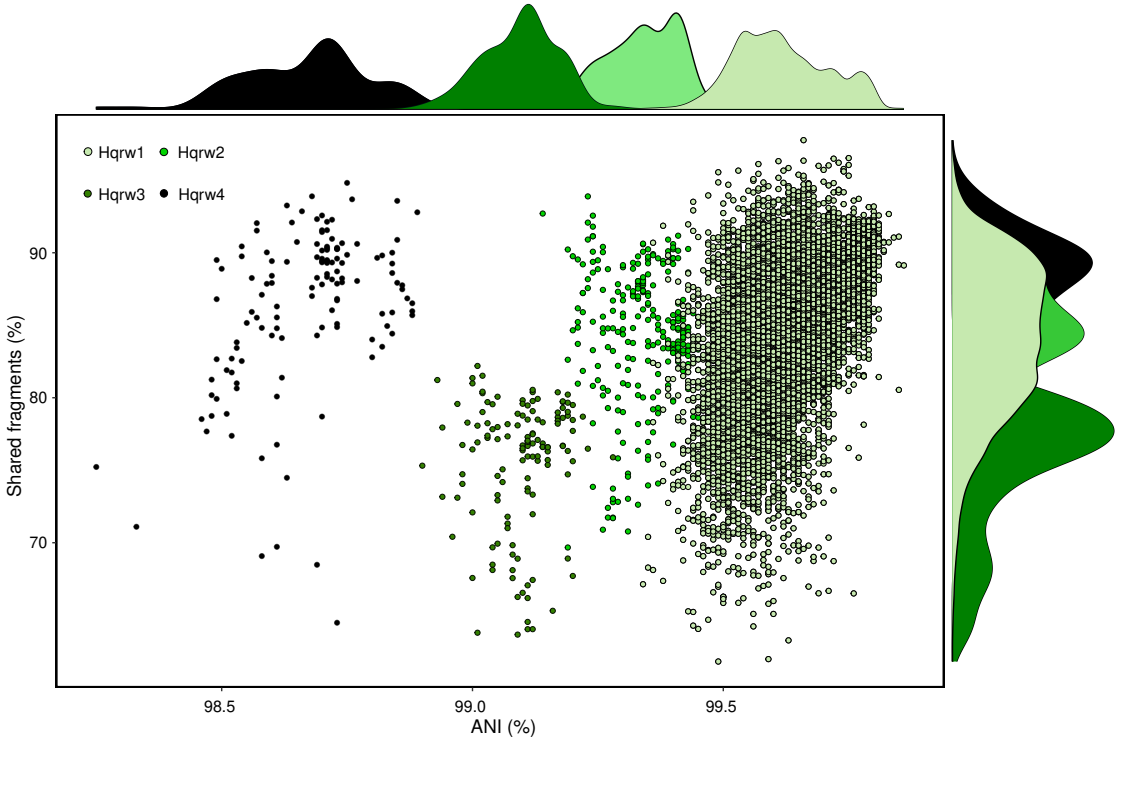
​​

**Supplementary Figure S6:** Comparison of intraspecies sequence diversity (ANIr) of HSP *Hqr. walsbyi* populations. ANIr values were estimated using the MAG recovered in each sample.


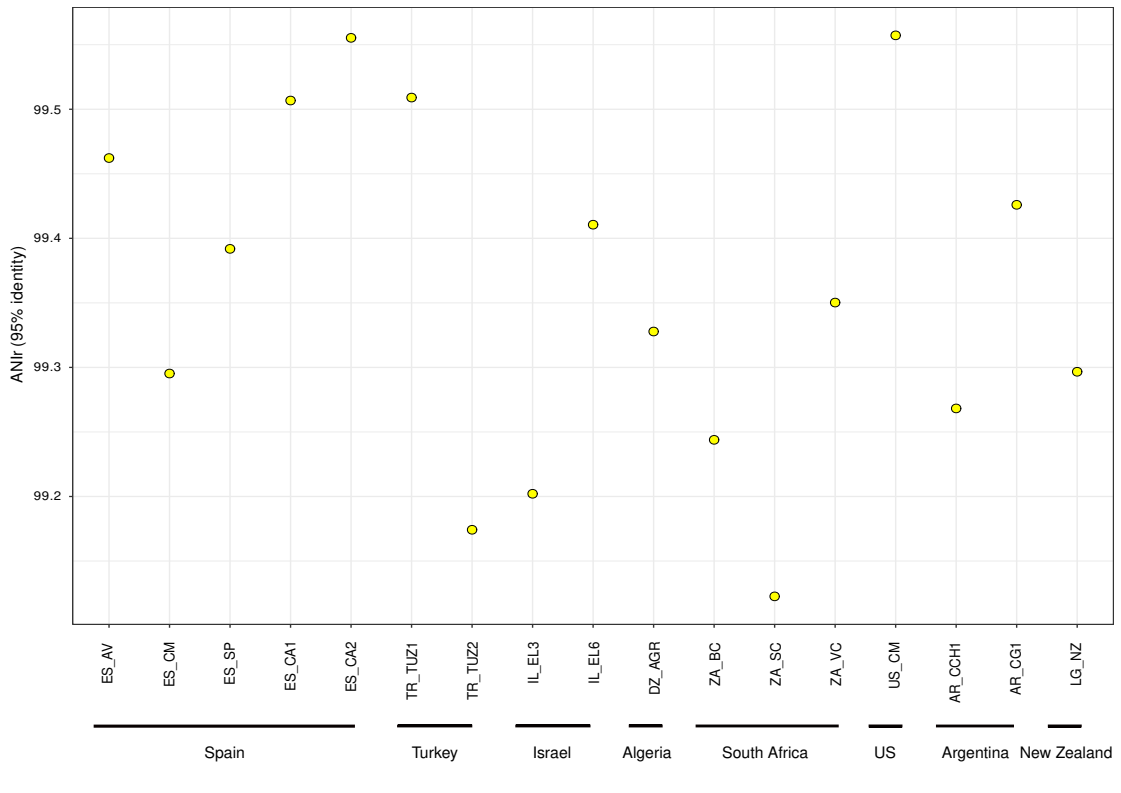


**Supplementary Figure S7:** NMDS based on ionic composition (g/L) of the HSP samples, including the following anions and cations: Cl^-^, Na^+^, Mg^2+^, SO_4_^2-^, K^+^, Br^-^, Ca^2+^, NO_2_^-^, Li^+^, NH_4_^+^, F^-^, PO_4_^3-^.


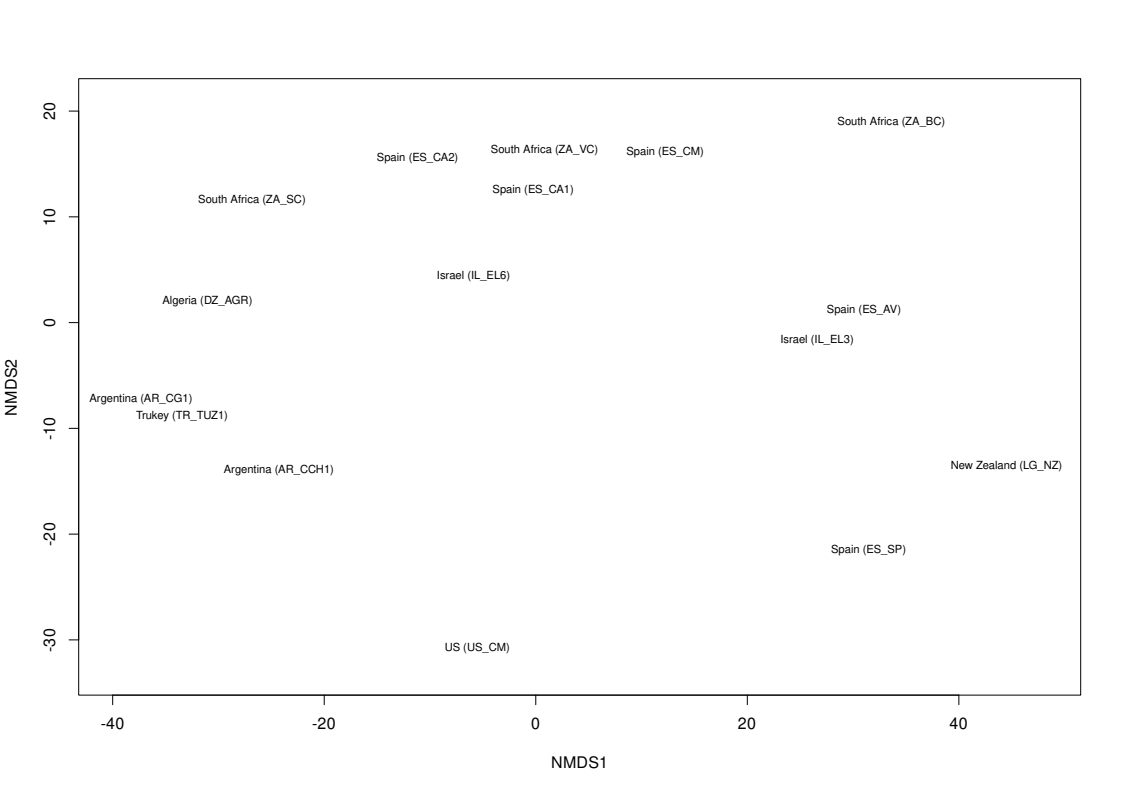


**Supplementary Figure S8:** Pangenome curves and metrics of the 142-genome collection of *Hqr. walsbyi* after 1,000 permutations*.* The top panel shows the three curves and their metrics based on the total number of non-redundant genes conforming the pangenome, the core genes and the number of specific genes. The bottom panel shows the gene per genome ratio and their metrics.

​​
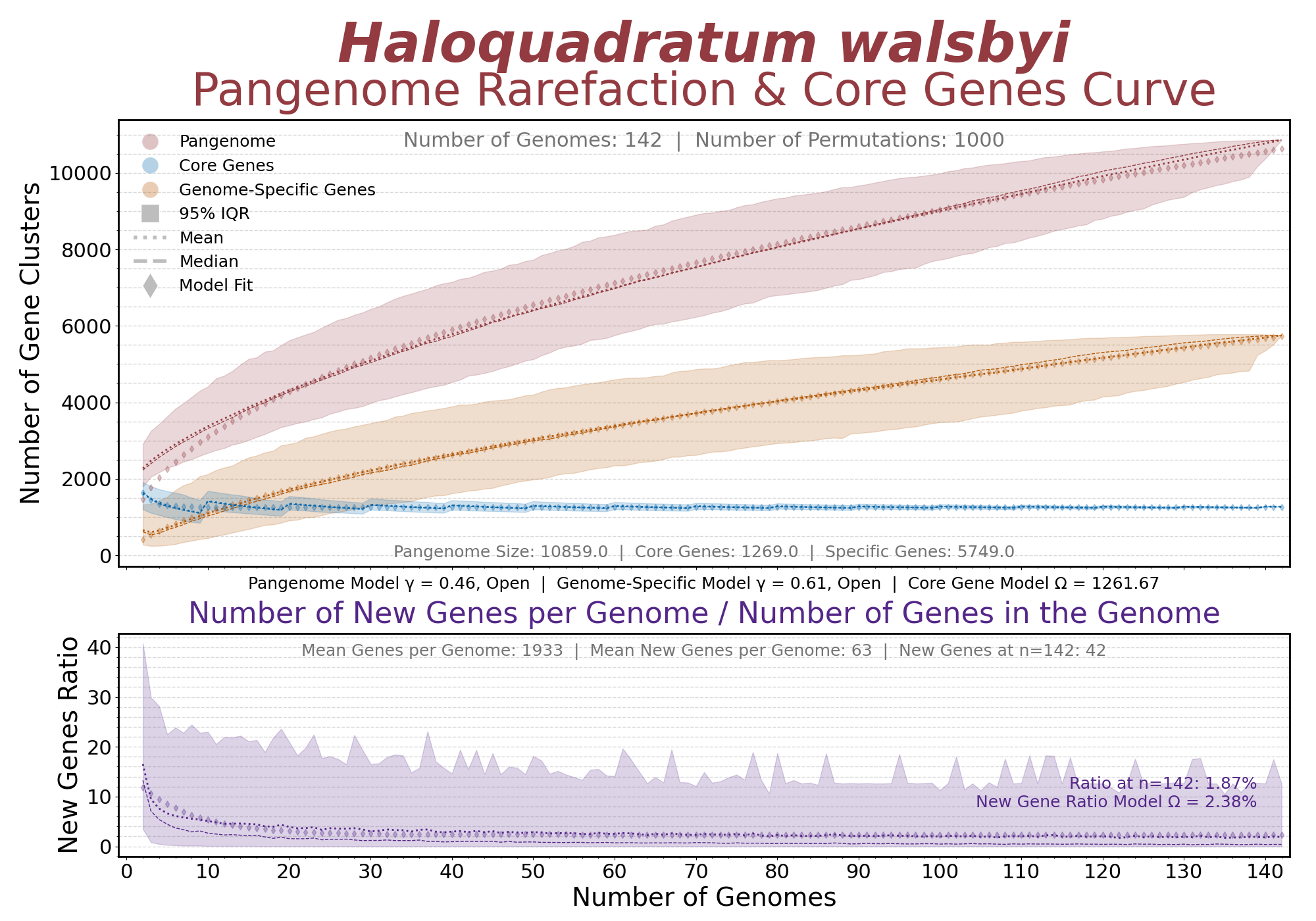
​​

**Supplementary Text S1: Functional comparison among genomovars and locations**

To further understand differences between genomovars and locations, we compared the genetic diversity of the 142 of *Hqr. walsbyi* MAGs and genomes collection. This dataset revealed an open pangenome (γ = 0.46; γ is the parameter that reflects the slope of the rarefaction curve representing the total non-redundant genes; Conrad et al., 2021), consisting of 10,859 non-redundant genes (Supp. Figure S8; Supp. Table S2). This pangenome comprised 1,269 core genes (11.69%), 1,239 auxiliary genes (11.41%), and 8,351 genomovar-specific genes (76.9%) (Supp. Table S2). Due to uneven representation, 94.06% of the genomovar-specific genes (7,855) were assigned to Hqrw1, while Hqrw2, Hqrw3, and Hqrw4 contributed 90, 240, and 167 unique genes, respectively (Supp. Figure S9; Supp. Table S2).

Functional annotations showed that 5,220 (66.46%) of Hqrw1-specific genes lacked matches in the TrEMBL database (i.e. hypothetical). Among the genes we observed features as a unique halomucin gene and numerous genes involved in membrane maintenance under osmotic stress (e.g. glycosyltransferases, major surface glycoproteins), transcriptional regulators, gas vesicles, universal stress proteins, and proteases (Supp. Figure S9; Supp. Table S2). Hqrw1 also encoded ~300 distinct ABC transporters targeting various organic (e.g. amino acids, peptides, carbohydrates, molybdenum, spermidine, phosphonate, urea) and inorganic (e.g. phosphate, potassium, sodium, metals, nitrate/nitrite, ferric, calcium) solutes (Supp. Figure S9; Supp. Table S2). A high number of mobile elements, including transposases, integrases/recombinases, and putative plasmid-related proteins, were also detected in this genomovar (Supp. Figure S9; Supp. Table S2). In Hqrw2, 52 hypothetical proteins (57.78%) were identified, alongside one exclusive halomucin gene, several transcriptional regulators, and genes involved in metabolism of coenzymes, amino acids, nucleotides, and carbohydrates (Supp. Figure S9; Supp. Table S2). According to the largest potassium concentration in Eilat salterns (11.1 g/L; Supp. Table S1), Hqrw2 contained transporters for potassium and other ions such as chromate and phosphate (Supp. Figure S9; Supp. Table S2). Hqrw3 encoded 213 hypothetical proteins (88.75%), one chemotaxis protein, diverse glycoproteins, and mobile elements such as transposases and integrases (Supp. Figure S9; Supp. Table S2). Aligning with the largest phosphate concentration (0.09 g/L; Supp. Table S1) observed in Colorada Chica (Argentina), Hqrw3 contained five phosphate transport-related proteins (e.g. PhoU, PstA, PstB) (Supp. Figure S9; Supp. Table S2). The most distinct genomovar, Hqrw4, had the fewest hypothetical proteins (n = 78; 46.71%) and was enriched in CRISPR-Cas systems, transposases, type I restriction-modification systems, and membrane-associated genes (e.g. glycoproteins, glycosyltransferases), but none specific transporters were identified (Supp. Figure S9; Supp. Table S2).

As Hqrw1 was detected in geographically diverse environments, we also investigated the differences within this genomovar by analyzing the 2,222 genes that were specific to each individual location of HSP (i.e. not including mesocosm MAGs) (Supp. Figure S9; Supp. Table S2). Supporting the high genomic similarity within *Hqr. walsbyi* and Hqrw1, most MAGs contributed very few unique genes: one in S’Avall (Mallorca; Spain), two in Veldriff (South Africa), six in Algeria, six in New Zealand, and 11 in Colorada Grande (Argentina), and 38 in Great Salt Lake (US), mostly annotated as hypothetical (Supp. Figure S9; Supp. Table S2). In addition, no unique genes were found in the MAG from Es Trenc (Mallorca; Spain) (Supp. Figure S9; Supp. Table S2). Conversely, salterns with the most distinct ionic profiles, such as those in the Canary Islands, Santa Pola, Soutpan, Bergpan, and Turkey, harbored the highest numbers of location-specific genes in Hqrw1, ranging from 187 to 963 (Supp. Figure S9; Supp. Table S2). MAGs from Turkey contained 963 unique genes, including 573 hypothetical proteins (59.5%), ABC transporters (e.g. for phosphate and calcium), membrane-related proteins (e.g. glycosyltransferases, glycoproteins, polysaccharide biosynthesis), and CRISPR-Cas proteins (Supp. Figure S9; Supp. Table S2). Canary Island MAGs had 520 unique genes, 322 of which were hypothetical (61.92%), along with exclusive ABC transporters (not phosphate-related), membrane-associated proteins, and CRISPR-Cas systems (Supp. Figure S9; Supp. Table S2). Santa Pola MAG and isolate showed 196 specific genes, including 90 as hypothetical (45.92%) and several transporters, transposases, integrases, and glycosyltransferases (Supp. Figure S9; Supp. Table S2). Focusing on inland salterns of South Africa, we detected 297 location-specific genes for Bergpan, which 184 of them were identified as hypothetical (61.95%), along with several membrane maintenance genes, transposases, and ABC transporters (Supp. Figure S9; Supp. Table S2). Similarly, Soutpan contained 187 genes, which largely were not assigned to any function (n = 93; 43.73%), accompanied by ABC transporters related to both organic (e.g. amino acid, amide) and inorganic (e.g. phosphate, urea) solutes, glycosyltransferases (e.g. AglB, AglG, WbuB), and mobile elements (Supp. Figure S9; Supp. Table S2).

**Supplementary Figure S9:** Presence and absence heatmap based on the number of non-redundant genes comprising the pangenome of *Hqr. walsbyi* comparing between **A)** genomovars and **B)** locations.


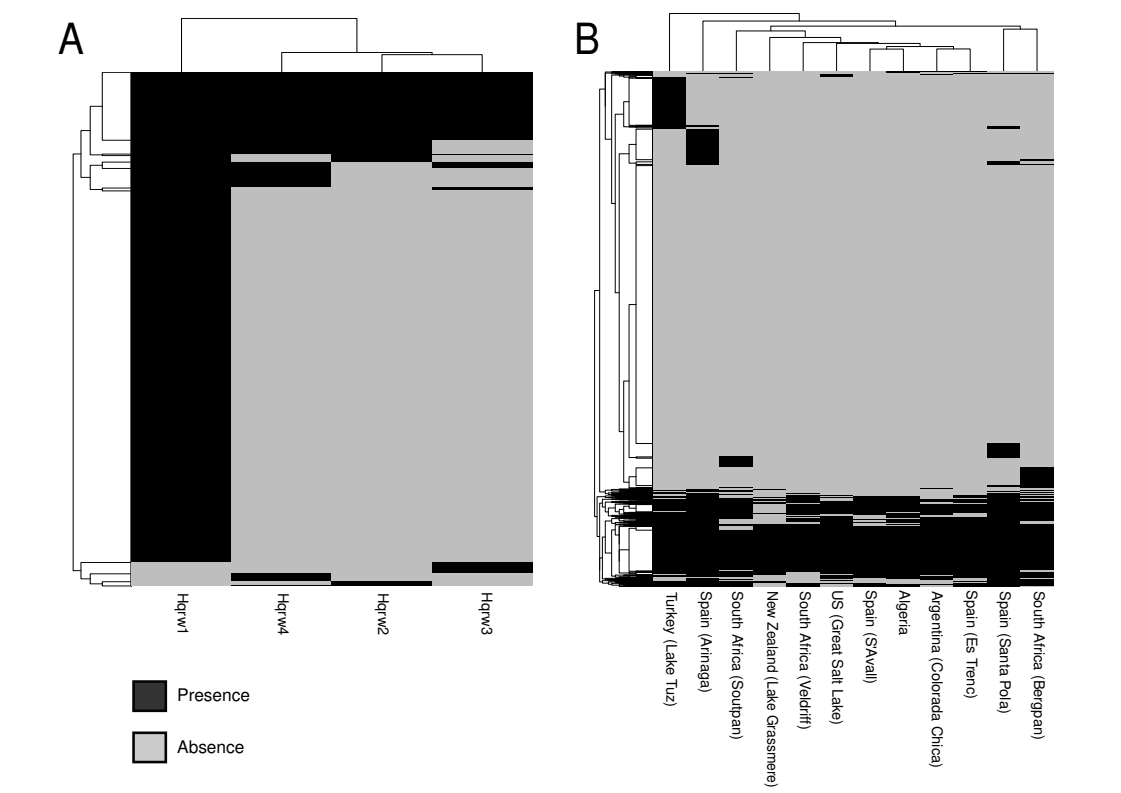


**Supplementary Figure S10:** Line plot representing the percentage of reads mapping at 99.3% identity to the four-genomovar collection compared to the total species abundance (i.e. 95% identity reads).


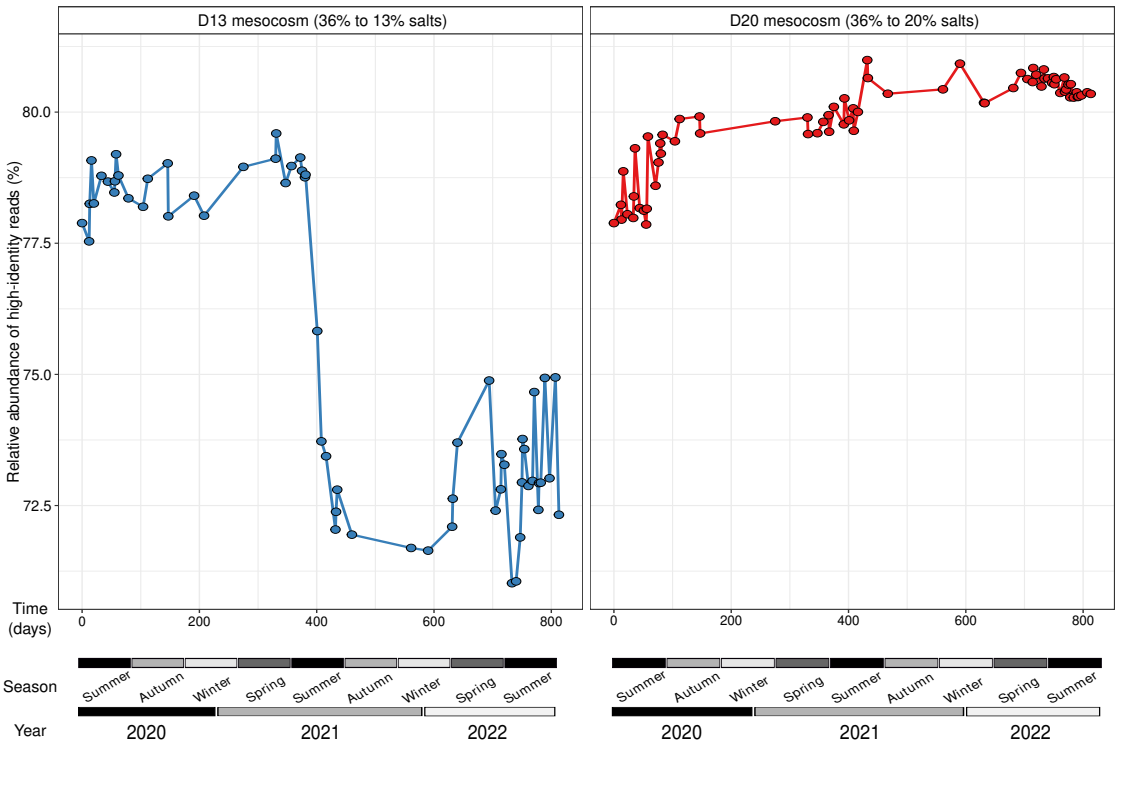


**Supplementary Figure S11:** Heatmap showing the relative abundance of the 8,067 vOTUs viral abundances at both mesocosm and global scales. vOTUs abundances were calculated based on sequencing effort (sequencing depth divided by number of metagenomic reads) across metagenomes (n=130).


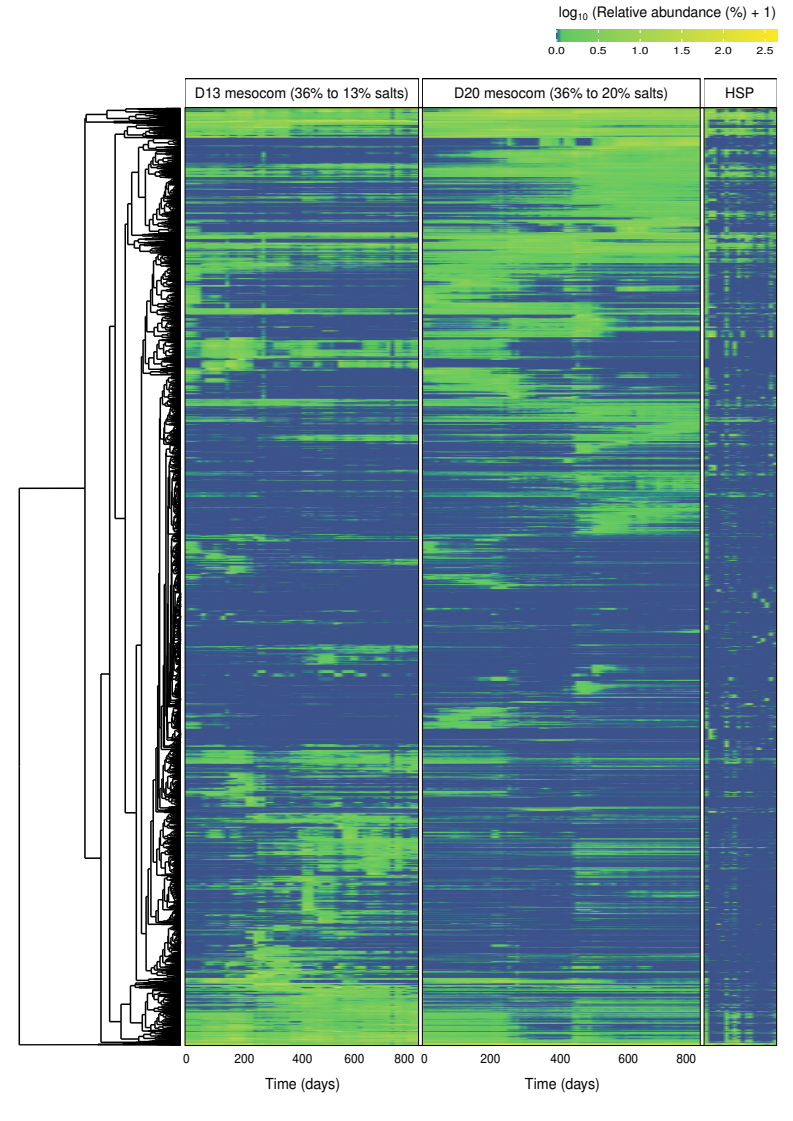


**Supplementary Figure S12:** Line plot representing the relative abundance of the 639 vOTUs predicted to infect *Hqr. walsbyi* in both D13 and D20 mesocosms. Relative abundances of viruses were estimated using sequencing effort (i.e. sequencing depth divided by the total number of reads in the metagenome and multiplied by 10^8^).


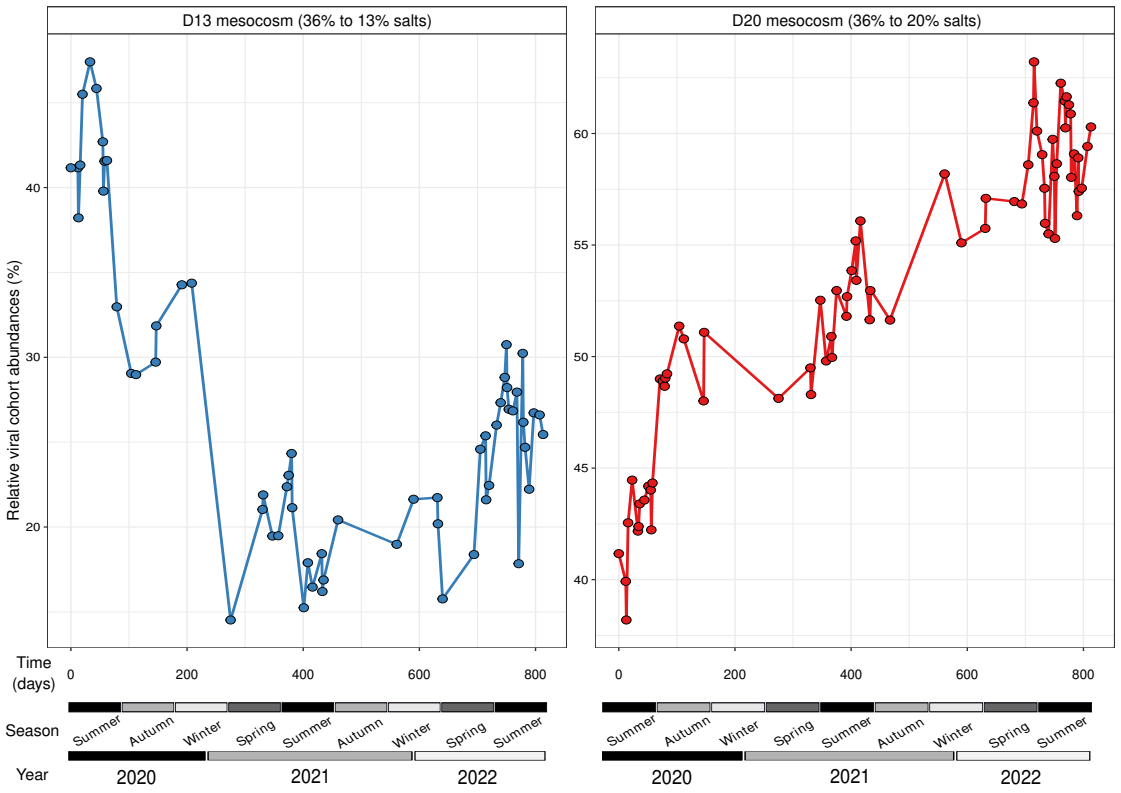


**Supplementary Figure S13:** Barplot representing the relative abundance of the 639 vOTUs predicted to infect *Hqr. walsbyi* in HSP. Relative abundances of viruses were estimated using sequencing effort (i.e. sequencing depth divided by the total number of reads in the metagenome and multiplied by 10^8^).


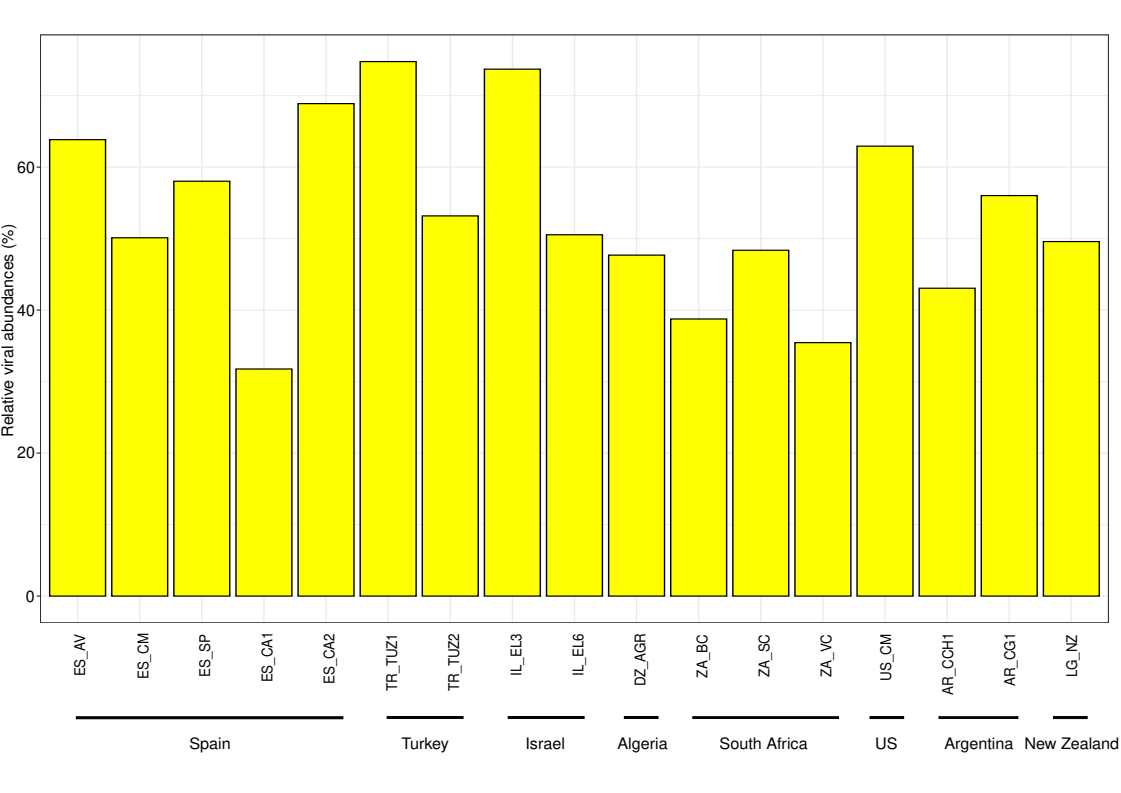

Supplement: Bustos-Caparros_Supp_Material_Third_Resubmission_wraf165 [file bustos-caparros_supp_material_third_resubmission_wraf165.docx]
